# Synthesis and defect characterization of hybrid ceria nanostructures as a possible novel therapeutic material towards COVID-19 mitigation

**DOI:** 10.1038/s41598-022-07200-9

**Published:** 2022-02-28

**Authors:** L. S. R. Rocha, A. Z. Simões, C. Macchi, A. Somoza, G. Giulietti, M. A. Ponce, E. Longo

**Affiliations:** 1 Center for Research and Development of Functional Materials, Federal University of São Carlos (UFSCar), São Carlos, SP Brazil; 2 School of Engineering, São Paulo State University (UNESP), Guaratinguetá, SP Brazil; 3 CIFICEN (UNCPBA-CICPBA-CONICET) and Instituto de Física de Materiales Tandil (UNCPBA), Pinto 399, B7000GHG Tandil, Argentina; 4 National University of Mar del Plata (UNMdP), Mar del Plata, Argentina

**Keywords:** Nanoscale materials, Nanoscale materials

## Abstract

This study reports the synthesis of hybrid nanostructures composed of cerium dioxide and microcrystalline cellulose prepared by the microwave-assisted hydrothermal route under distinct temperature and pH values. Their structural, morphological and spectroscopic behaviors were investigated by X-Rays Diffraction, Field Emission Gun Scanning Electron Microscopy, High-Resolution Transmission Electron Microscopy, and Fourier-Transform Infrared, Ultraviolet–Visible, Raman and Positron Annihilation Lifetime spectroscopies to evaluate the presence of structural defects and their correlation with the underlying mechanism regarding the biocide activity of the studied material. The samples showed mean crystallite sizes around 10 nm, characterizing the formation of quantum dots unevenly distributed along the cellulose surface with a certain agglomeration degree. The samples presented the characteristic Ce–O vibration close to 450 cm^−1^ and a second-order mode around 1050 cm^−1^, which is indicative of distribution of localized energetic levels originated from defective species, essential in the scavenging of reactive oxygen species. Positron spectroscopic studies showed first and second lifetime components ranging between 202–223 ps and 360–373 ps, respectively, revealing the presence of two distinct defective oxygen species, in addition to an increment in the concentration of Ce^3+^-oxygen vacancy associates as a function of temperature. Therefore, we have successfully synthesized hybrid nanoceria structures with potential multifunctional therapeutic properties to be further evaluated against the COVID-19.

## Introduction

The coronavirus family, which includes SARS-CoV-2, has caused three pandemics in the last 18 years [SARS-CoV (2003), MERS-CoV (2012), COVID-19 (2019)]^1^⁠, with the latter emerging as an unprecedented global health crisis^2^⁠ responsible for more than 200 million confirmed cases and 4 million deaths, according to the World Health Organization (WHO).

In this way, the nanomedicine field with its promising candidates forming an interface with pathogenic viruses to mitigate their adverse effects using nanomaterials comprising functionalized nanoparticles (NPs) and quantum dots (QDs) have to be considered. Thus, the design of potential nanomedicines based on eco-friendly nanostructured materials for diagnosis, prevention, and the combat of these diseases becomes urgent. As a result, several strategies have been proposed in the context of the COVID-19 pandemic^3^⁠ as complementary solutions to the vaccine’s development.

Besides, worth mentioning the importance of nanomaterials and hybrid nanostructures in their wide range of applications, such as the Dy_2_Sn_2_O_7_ nanostructures prepared by an environmentally friendly route and applied as visible-light-sensitive photocatalyst for the destruction of organic contaminants in water^4^⁠, as well as the polycarbonate (PC)-polyurethane (PU) membranes blended with alumina (Al_2_O_3_) nanoparticles for removal of humic acid molecules^5^⁠. Besides, worth mentioning the innovative approach of a controllable combustion method used to prepare Zn–Co–C nanocomposites with new and green reactants for the removal of acid brown 14 under sunlight^6^⁠. Additionally, we can mention the application of nanostructures for energy storage, as the Nd_2_Sn_2_O_7_ with a discharge capacity after twenty cycles of 4013 mAh/g (~ 14.2 wt%)^7^⁠.

Since the pioneering work of Zholobak et al.^8^⁠, who demonstrated the antiviral ability of polymer-coated ceria nanoparticles, many researchers have been intensively studying the antiviral properties of nanoceria. As the first lanthanide element, cerium has a 4f. electronic configuration and a unique cubic fluorite structure with vacant oxygen in its lattice that allows the alternation between Ce(III) and Ce(IV) states. This is a key characteristic when it comes to nanoceria (NC) emerging as a remarkably safe, redox regenerative, rare-earth-based nanomedicine against acute and chronic inflammatory diseases characterized by redox imbalances and reactive oxygen species (ROS), such as Alzheimer’s disease, Parkinson’s disease, diabetes mellitus and a variety of cancers^9–12^.

Nanoceria has been considered a robust antioxidant with catalase and superoxide dismutase mimetic activity^13,14^ that contributes to the neutralization of ROS and reactive nitrogen species, besides considerable pharmacological relevance due to its regenerative antioxidant potential as a result of the redox cycling between Ce^+3^ and Ce^+4^ ^15,16^. On the other hand, it has been reported to reduce fibrogenic signaling by the inhibition of the TGF-β signaling pathway^17^⁠ and to combat acute inflammatory insult, reducing severe sepsis-related mortality through the inhibition of NFkB signaling and the suppression of lipopolysaccharide-induced MAPK signaling^18,19^, which are key contributors to complications related to COVID-19. Manne et al. showed that NC can be successfully used against peritonitis, proving its effective anti-inflammatory activity^20^⁠.

Regarding the SARS-CoV-2 inactivation mechanism, it is known that inflammatory cells like neutrophils and macrophages actively participate in the pathological response, with cytokines such as interleukin (IL)-1β, IL-6, IL-8, tumor necrosis factor (TNF)-α and TGF-β playing a fundamental role in the pathogenesis of COVID-19. The synthesis and release of cytokines, triggered by pathogenic viral stimuli, induce IkB kinase (IKK) and c-Jun N-terminal kinase (JNK) protein complex, which further stimulates the production of excessive cytokines by amplifying the transcriptional activity of NFkB and activator protein 1 (AP1)^21^⁠.

In this context, nanoceria has been shown to attenuate the cytokine storm by affecting the autocrine and paracrine pathways, directly decreasing or suppressing the cytokine synthesis by blocking the receptor interaction of cytokines through the modulation of p65-NFkB, MAP kinase/NFkB and Nrf2/NFkB pathways^22,23^. Therefore, nanoceria can be used as a novel therapeutic material for the management of COVID-19 through either direct virus inactivation on surfaces or administration in the body, interrupting the progression of systemic inflammatory complications due to its ability to inhibit NFkB, MAPKs, and TGF-β signaling pathways^24^⁠.

Unfortunately, free ceria nanoparticles administered in the body can be easily dissolved, forming toxic species, or even excreted from the organism^25^⁠. Thus, improved strategies must be developed to enhance their performance, for example, to bind them to a polymeric matrix in order to improve their antioxidant properties and decrease their toxicity, as demonstrated by Weaver et al.^26^⁠. Therefore, the design of ceria-based hybrid nanostructures would not only allow the modulation of the anti-/pro-oxidant activity of CeO_2_ nanoparticles, but also enhance their antimicrobial and antioxidative properties^27^⁠. Biocompatibility and toxicity data from experimental observations provided by Kalyanaraman et al.^28^⁠ showed a solid base through a 28-day systemic toxicity and genotoxicity survey performed according to current regulatory standards, displaying very low local tissue reactions (implantation irritation index of less than 3), and thus better toleration than most other implant materials tested. Furthermore, NC virtually demonstrated no systemic toxicity or in vivo micronucleus induction in bone marrow via the implantation route.

In terms of synthesis routes, several physical/chemical processes have been reported for the preparation of CeO_2_ nanoparticles (NPs), such as sol–gel, precipitation, ball milling, hydrothermal decomposition, thermal decomposition, pyrolysis and hydrolysis among others^29,30^. Worth mentioning the simple and surfactant-free sonochemical pathway used to prepared pure and copper (Cu)-doped (4 and 40 wt%) CeO_2_ nanostructures for hydrogen storage purposes, with the 4 wt% sample depicting a discharge capacity of 5070 mAh/g at 22nd cycle^31^⁠, as well as the facile and eco-friendly route used to prepare Pr_2_Ce_2_O_7_ nanostructures from banana extract^32^⁠ and the use of grape juice as a novel and green fuel to obtain cobalt ferrite nanocomposites^33^ However, none of them enables the preparation of highly crystalline nanostructured materials with low temperature and reaction times (100 °C, 8 min)^34^⁠ like the microwave-assisted hydrothermal (MAH) synthesis. Besides, common techniques for the preparation of metal oxide-polymer nanocomposites include^35^⁠: (1) polymerization of monomers in the presence of metal oxide nanoparticles in situ; (2) direct blending of metal oxide nanoparticles and polymers by mixing in a melt or solution; and (3) the sol–gel process.

Regrettably, there are only a few reports on the synthesis of cellulose-modified nanoceria and even fewer results aiming at antibacterial/virucide properties. For instance, in one study a multifunctional linen fabric modified with CeO_2_ was prepared, showing highly effective activity against *S. aureus* and *E. coli,* with most of the functional properties retained at a satisfactory level after five subsequent washes^36^⁠. In another work, CeO_2_ nanoparticles were synthesized by the sol–gel method using MCC as a template and a specific surface area 5.5 times higher than that of the pure ceria for the catalytic ozonation of phenol, resulting in a removal efficiency of 69.2% and 49.5% for the modified and pure systems, respectively^37^⁠. In contrast, Ag/cellulose nanocrystal-doped CeO_2_ QDs were prepared by the co-precipitation method for the degradation of methylene blue and ciprofloxacin (MBCF), reaching a 99.3% degradation with a concentration of 4% Ag dopant^38^⁠.

Regarding the evaluation of the electronic structure and the surface composition of semiconductor QDs such as nanoceria, the Positron Annihilation Spectroscopy (PAS) has proved to be a highly sensitive technique^39–42^. However, whether the confinement of the positron wave function is inside the QDs or localized at their surfaces is still an open question. For the first time, Weber et al.^39^⁠ used different experimental variants of PAS to study colloidal CdSe QDs sized from 6 to 1.8 nm. The authors specifically used Coincidence Doppler Broadening (CDBS) and Positron Annihilation Lifetime (PALS) Spectroscopies and stated that at least for the studied system positrons annihilate into the quantum structures and that a size-dependent signature is imprinted onto the annihilation radiation. On the other hand, although studying the same system Eijt et al.^40^⁠ proposed that positrons were mainly located at the surfaces of CdSe QDs. These authors described the positron wave function as a ‘shell-like’ state at the QD surface. Recent PALS measurements on PbSe QDs have indicated the existence of a positron surface state in these QDs^43^⁠. Additionally, PALS has been used to study the presence of microstructural defects in La-doped ZnO QDs and their relationship with QD sizes^42^⁠. Additionally, a similar study on TiO_2_-sensitized multi-sized CdTe QDs was carried out^44^⁠.

In this context, we used the MAH route as a direct blending alternative to synthesize hybrid structures composed of NC with a polymeric matrix of microcrystalline cellulose (MCC) and performed microstructural, morphological, and spectroscopic characterizations using XRD, FEG-SEM, HR-TEM and FT-IR, UV–Vis, Raman and Positron Annihilation Lifetime Spectroscopies to investigate the Ce(III)/Ce(IV) redox cycling and the polymer decoration capability. Considering the group’s expertise in the application of chemical finishing onto fabrics aiming at^45^⁠ SARS-CoV-2 inactivation, future evaluations with the CeO_2_@MCC hybrid systems will also be performed.

## Experimental procedures

The hybrid nanostructures composed of CeO_2_ and microcrystalline cellulose (MCC) were prepared by the microwave-assisted hydrothermal (MAH) route at 100 and 120 °C to investigate the influence of temperature on the nanoceria crystals. Firstly, a 0.5 M cerium nitrate hexahydrate [Ce(NO_3_)3.6H_2_O, Sigma Aldrich, 99%] solution was prepared in distilled water, followed by the addition of 25 wt% of microcrystalline cellulose (Synth, 99%). Secondly, we took equal volumes for pH adjustments to 10, 12, and 14 [2 M potassium hydroxide (KOH, 99.5%, Labsynth)]. Then, we took aliquots for two separate syntheses (100 °C and 120 °C) under a microwave frequency of 2.45 GHz (Panasonic NN-ST357WRP, 800 W) at a heating rate of 10 °C/min for 8 min. The obtained solutions were then centrifuged at 2000 rpm for 10 min, which was repeated for 3 times, and dried in a laboratory oven at 100 °C for 24 h. The as-synthesized powders were characterized by X-Rays Diffraction (XRD) using a Rigaku-Dmax/2500 PC (UFSCar, São Carlos, SP) with a Cu-Kα radiation λ = 1.5406 Å in the 2θ range of 20–80° at room temperature. High-resolution Transmission Electron Microscopy (HR-TEM FEI Tecnai G2-20, 200 kV) and Field-Emission Gun Scanning Electron Microscopy (FEG-SEM Supra 35-VP) (UFSCar, São Carlos, SP) were used to observe the morphology and size of the as-synthesized particles as well as their distribution along the microcrystalline cellulose surface. Ultraviolet–visible (UV–Vis) spectra were collected using a Varian Cary 5G (UFSCar, São Carlos, SP) in diffuse reflectance mode in the wavelength range of 200–800 nm. The effective band gap (Eg) energy was obtained by means of a Tauc plot. The Fourier-Transform Infrared (FT-IR) spectra were recorded with a Rayleigh spectrometer model WQF-510A (UFSCar, São Carlos, SP) in transmittance mode. The Raman spectroscopy characterization was obtained on a LabRAM iHR550 Horiba Jobin Yvon spectrometer (UFSCar,São Carlos, SP) with spectral resolution of 1 cm^−1^ and 40 scans in the range of 200–1500 cm^−1^, coupled to a CCD detector. The procedure was carried out using an argon-ion laser with a wavelength of 514.5 nm and a power of 8 mW.

PALS spectra were obtained using a fast–fast system with a time resolution of 275 ps in a collinear geometry. As a positron source, a 10 µCi sealed source of ^22^NaCl deposited onto two thin Kapton foils (7.5-µm thick) sandwiched between two identical samples was used. The spectra were acquired at RT, and typically 1.5–2 × 10^6^ counts per spectrum were collected. The lifetime values reported in this work for each sample are at least an average of 10 measurements in the same experimental conditions. After subtracting the background and the source contribution, the positron lifetime spectra were analyzed using the LT10 code^46^⁠.

## Results and discussion

### X-rays diffraction (XRD)

**Figure 1:**
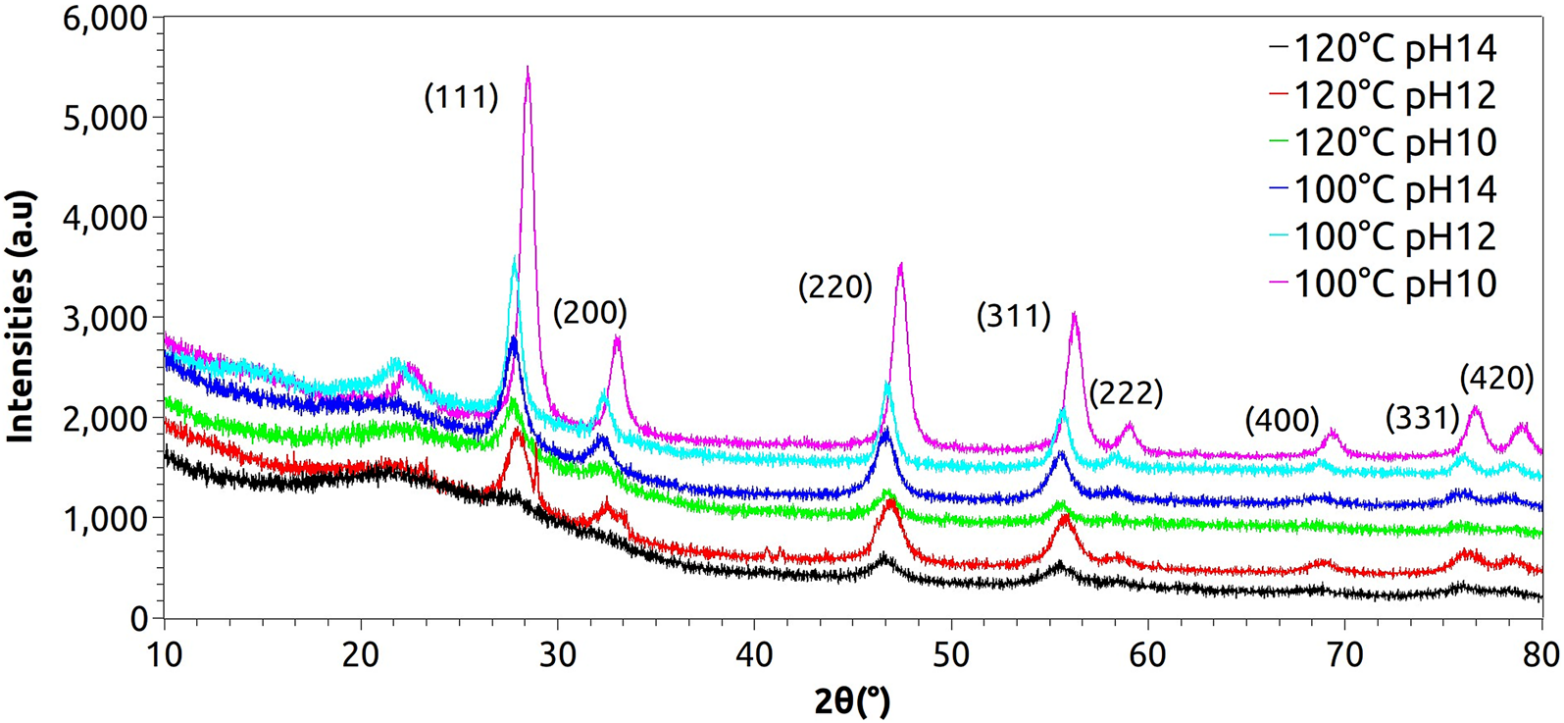
XRD patterns of the samples synthesized by microwave-assisted hydrothermal method at 100 and 120 °C with pH 10, 12 and 14.

The powder XRD of the as-prepared hybrid nanostructures showed similar patterns for all synthesis conditions, as seen in Fig. 1, with the signal around 22º indicating the presence of cellulose-type I crystalline structure^47,48^. The other main peaks can be clearly indexed to a fluorite-type cubic structure, according to the prominent diffraction of the [111] lattice planes characteristic of CeO_2_ (space group Fm3m), with theoretical lattice constant a = 5.411 Å (JCPDS 34-394). When comparing these results with the pure and Fe-doped CeO_2_ obtained by microwave-assisted combustion synthesis with evident formation of a secondary phase of CeFeO_3_^49^⁠ as well as to the micro and nanocrystalline CeO_2_ irradiated with 946 MeV Au ions at room temperature with the presence of a secondary phase of Ce_11_O_20_^50^⁠, the purity of our samples is confirmed.

It is possible to observe that the intensity of the main peak (111) decreases as the temperature and pH increase, generating local distortions in the lattice, increasing the full width at half maximum (β parameter) and reducing the mean crystallite size (D). Besides, a possible degradation of the cellulose crystalline backbone under harsh conditions (120 °C, pH 12 and 14) within the microwave environment likely contributes to this phenomenon, as noted by the deformation of the (111) signal and a broad-band formation around 22°.

The mean crystallite size (*D*) was calculated using the Scherrer equation as follows: (K.λ)/(β.cosθ), where K is the shape factor (we used 0.94 as a good approximation for cubic symmetries), λ is the X-ray wavelength, β is the full width at half maximum (FWHM) in radians obtained with the aid of open-source software QtiPlot (0.9.8.9 svn 2288, 02/11/2011) by a Lorentzian fit of the (111) peaks, and θ is the diffraction angle of the main peak^51^⁠, as seen in Table 1. The values are close to estimation found in the literature of 8.5 to 21.8 nm, reported by Eaimsumang, S. et al.^52^⁠, as well as the values of 20 and 15 nm for pure and Eu-doped CeO_2_, found by Gnanam, S. et al.^53^⁠. This table also presents the experimental lattice constants (a = b = c) for the (111) diffraction peaks, which are very close to the theoretical value of 5.411 Å.

**Table 1:** Debye–Scherrer mean crystallite sizes (D).

Temperature (°C)	pH	b (°)	2q_111_ (°)	a (Å)	*D* (nm)
100	10	0.684	28.45	5.43	12.51
	12	0.687	27.77	5.57	12.44
	14	1.02	27.69	5.56	8.37
120	10	1.30	27.69	5.56	6.57
	12	1.24	27.88	5.54	6.89
	14	4.55	27.76	5.56	1.88

Regarding the influence of temperature on the mean crystallite size, it is expected to grow with higher temperatures^54^⁠. However, our samples behaved differently, with a reduction in D with increasing temperature, suggesting the prevention of crystal growth^55^⁠. When compared to the pure ceria system prepared by the MAH route with mean crystallite size of 16.56 nm^56^⁠, the cellulose-modified systems presented reduced mean crystallite sizes, demonstrating that the MCC served as a template for more CeO_2_ nucleation^57^⁠. In terms of pH changes, very close values could be observed for the same temperature possibly indicating weak influence of pH on long-range order symmetries.

It is clear that the crystallites of the samples are close to 10 nm, suggesting the formation of CeO_2_ quantum dots (QD)^58^⁠, which would facilitate their administration via aerosol delivery for COVID-19 patients^59^⁠.

Therefore, our results corroborate the capability of the MAH technique to produce hybrid nanostructures with long-range order under short reaction times and temperatures.

### Field emission gun scanning electron microscopy (FEG-SEM)

**Figure 2:**
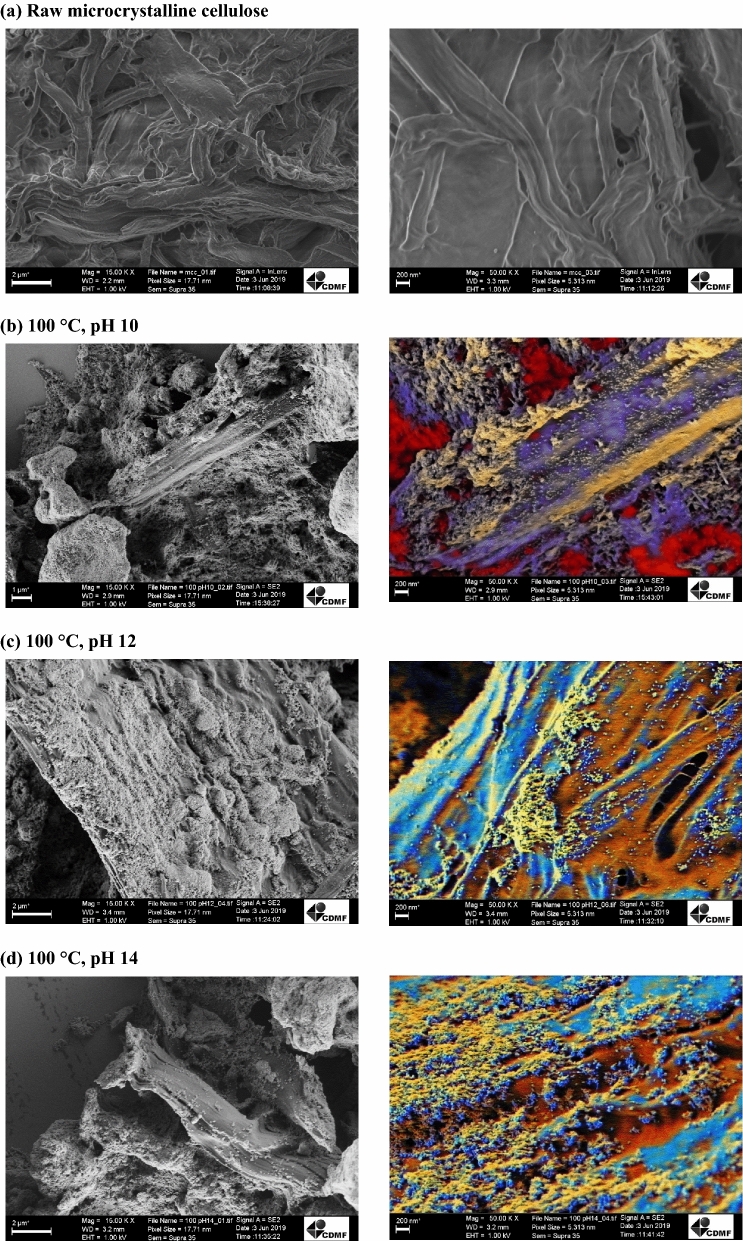

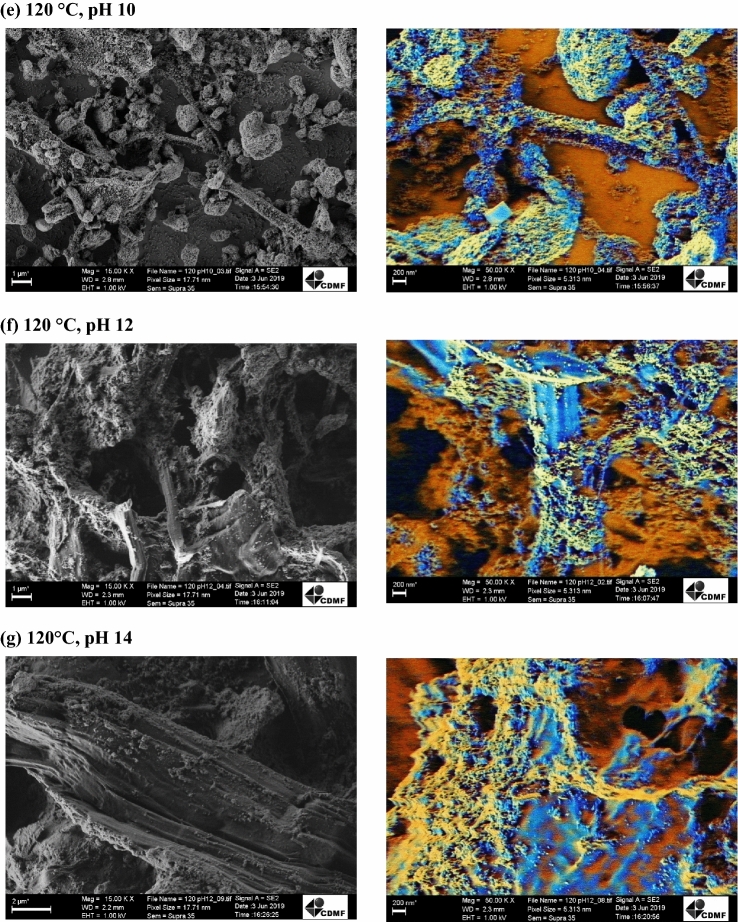
SEM micrographs of the (**a**) raw microcrystalline cellulose and hybrid structures prepared with the MAH route at (**b**) 100 °C, pH 10, (**c**) 100 °C, pH 12, (**d**) 100 °C, pH 14, (**e**) 120 °C, pH 10, (**f**) 120 °C, pH12 and (**g**) 120 °C, pH 14.

The morphologies of the CeO_2_/MCC hybrid structures were investigated by SEM and are shown in Fig. 2. It is possible to observe that the CeO_2_ crystals grow among the cellulose, with the ceria crystals forming nano-agglomerates throughout the cellulose surface due to their reduced size and strong Van der Waals interaction^34^⁠. It is worth mentioning that the original micrographs without digital treatments are available in the Supplementary Information.

The effect of different temperatures and pH values on the shapes of the nanocomposites was also investigated. It was observed that when the temperature was raised to 120 °C, the CeO_2_ crystals were dispersed on the cellulose matrix, with pH 10 seeming to be the most homogeneous condition for a proper distribution of particles along the surface. Magnified images of the samples on the right side of Fig. 2b–g show ceria aggregation on the cellulose surface for all conditions. One distinctive characteristic can be seen in Fig. 2e, that is, a clear squared crystalline morphology among the aggregates, resembling a fractal of the fluorite-type cubic structure typical of pristine CeO_2_^60^. The synthesis at 120 °C, especially for pH 12 and 14, seemed to be a harsh condition for the synthesis of these hybrid nanostructures, as demonstrated by some degraded cellulose fibrils and little particle nucleation throughout the surface. According to previous reports, the particles morphologies can be modeled by changing the mineralizer concentration, as in the case of rod, cubes, beans and hexagons obtained by Amoresi et al.^61^⁠ using 6, 0.6 and 0.06 M NaOH solutions. It is worth mentioning that distinct morphologies, as in the case of nanospheres, nanoparticles, nanorods, and flower-like microspheres obtained by Dong et al. gave rise to different properties when dealing with CO oxidation^62^⁠.

According to the SEM pictures, it can be concluded that the nanocomposites with CeO_2_ nanoparticles dispersed in the cellulose matrix were successfully synthesized, corroborating the XRD results. These results demonstrated that the synthesis conditions not only play an important role in the crystallinity of CeO_2_ crystals, but also have a significant influence on their morphologies, which are determined by nucleation-dissolution–recrystallization processes controlled by parameters such as time, temperature and mineralizer (OH) concentration^63,64^.

It is worth mentioning that multiple nucleation sites are formed at the beginning of the MAH synthesis, when Ce(OH)_x_ species are deprotonated and converted into CeO_2_. With low mineralizer concentrations, low temperature and short synthesis time, the dissolution–recrystallization rate is slow, leading to the formation of irregular spherical-shaped nanostructures, which is the case here. By raising the synthesis temperature, time or mineralizer molarity, the dissolution–recrystallization rate increases, and distinct morphologies can be obtained, as reported in^61,65^.

### High-resolution transmission electron microscopy (HR-TEM)

**Figure 3:**
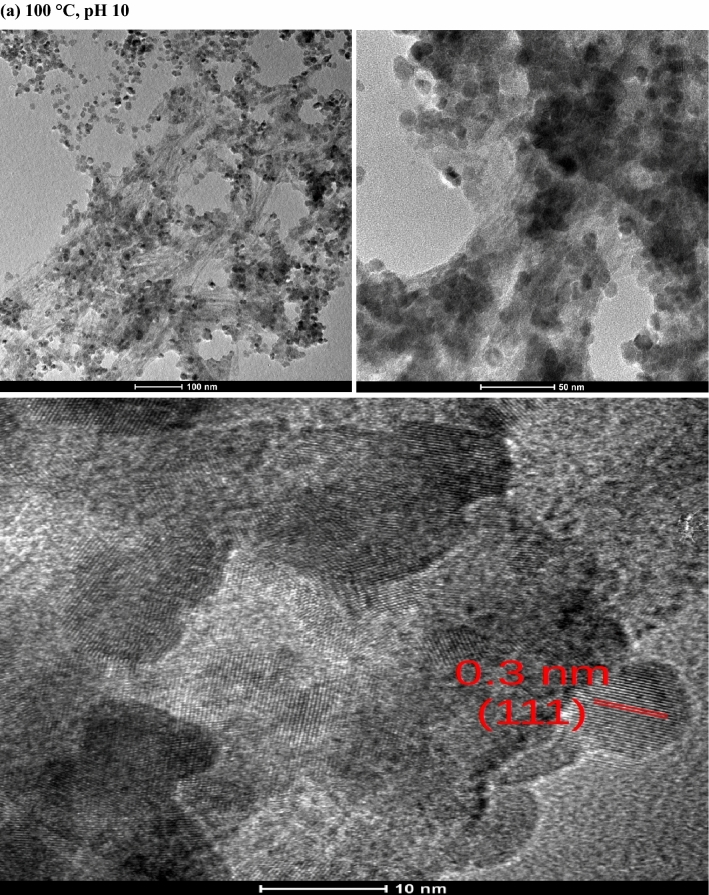

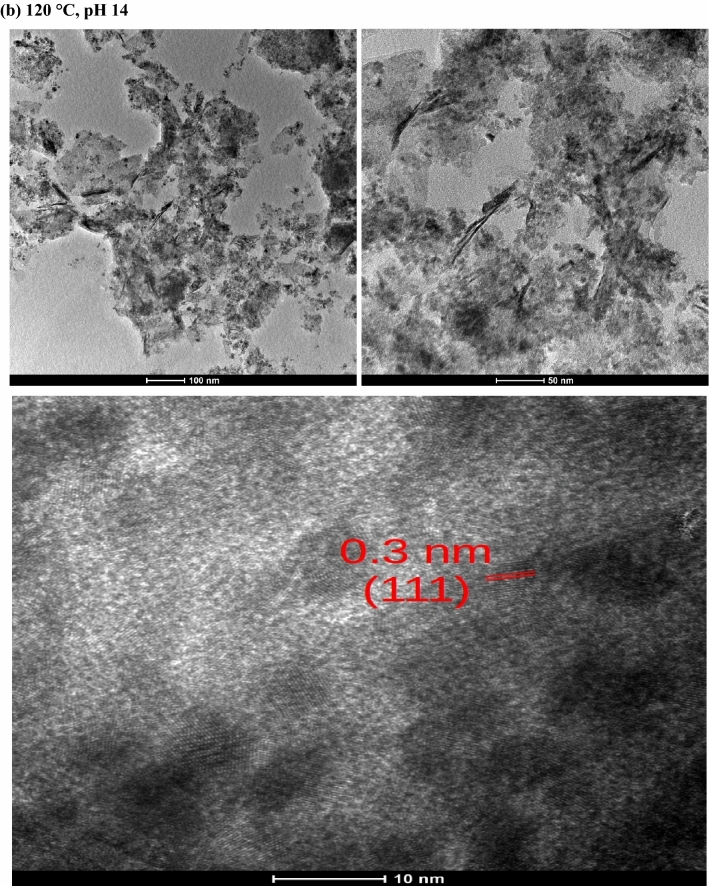
TEM micrographs of samples prepared at 100 °C and 120 °C with pH 10 and 14, respectively.

In order to estimate the size and morphology of the hybrid structures, the samples synthesized at 100 °C, pH 10 and 120 °C, pH 14 were investigated by high-resolution transmission electron microscopy (HR-TEM), as shown in Fig. 3. This figure reveals that the MAH product consists of unevenly dispersed quantum dots sized around 10 nm, distributed along the microcrystalline cellulose surface, close to the measured average crystallite sizes seen in Table 1. The interplanar spacing of both samples is around 0.3 nm, which matches the (111) fringes. It is interesting to note that Chen et al.^66^ have obtained different morphologies of CeO_2_ via a simple SO_2_ treatment with the rod-like CeO_2_ sample depicting an interplanar spacing of 0.31 nm, while the cube-like morphologies showed no (111) lattice fringes with an interplanar spacing of 0.26 nm, attributed to (200) crystal plane. Besides, Wei et al*.*^67^ has also obtained distinct morphologies (particles, rods and cubes) and investigated its effect on the Ni/CeO_2_ catalysts for selective hydrogenation of cinnamaldehyde, possessing interplanar spacings of 0.31 nm, which corroborates the results we found.

The rise of temperature and pH seems to have degraded some of the cellulose micro-fibrils (Fig. 3b), which appear with a darken contour if compared to Fig. 3a, corroborating the XRD results. Both samples present agglomeration due to Van der Waals forces, which is an indicative of ultra-fine particles with a tendency to form agglomerates in order to minimize their surface energy with a minimum surface-to-volume ratio^68^⁠ despite matching the interplanar space of the (111) fringes of a fluorite cubic structure.

Davoodbasha et al.^25^⁠ prepared a three-dimensional composite scaffold with cubic nanostructured particles sized between 3.2 and 32 nm made from cellulose and ceria nanoparticles by lyophilization, which was successfully immobilized into the cellulose matrix without agglomeration, besides presenting excellent antioxidant properties in a pH-dependent manner.

Therefore, this hybrid nanomaterial is a promising tool to be used in clinical applications as an effective antioxidative green material for scavenging reactive oxygen species, with the cerium valence states drastically affecting the cell behavior on the scaffold surface^69,70^.

### Ultraviolet–visible (UV–Vis) spectroscopy

**Figure 4:**
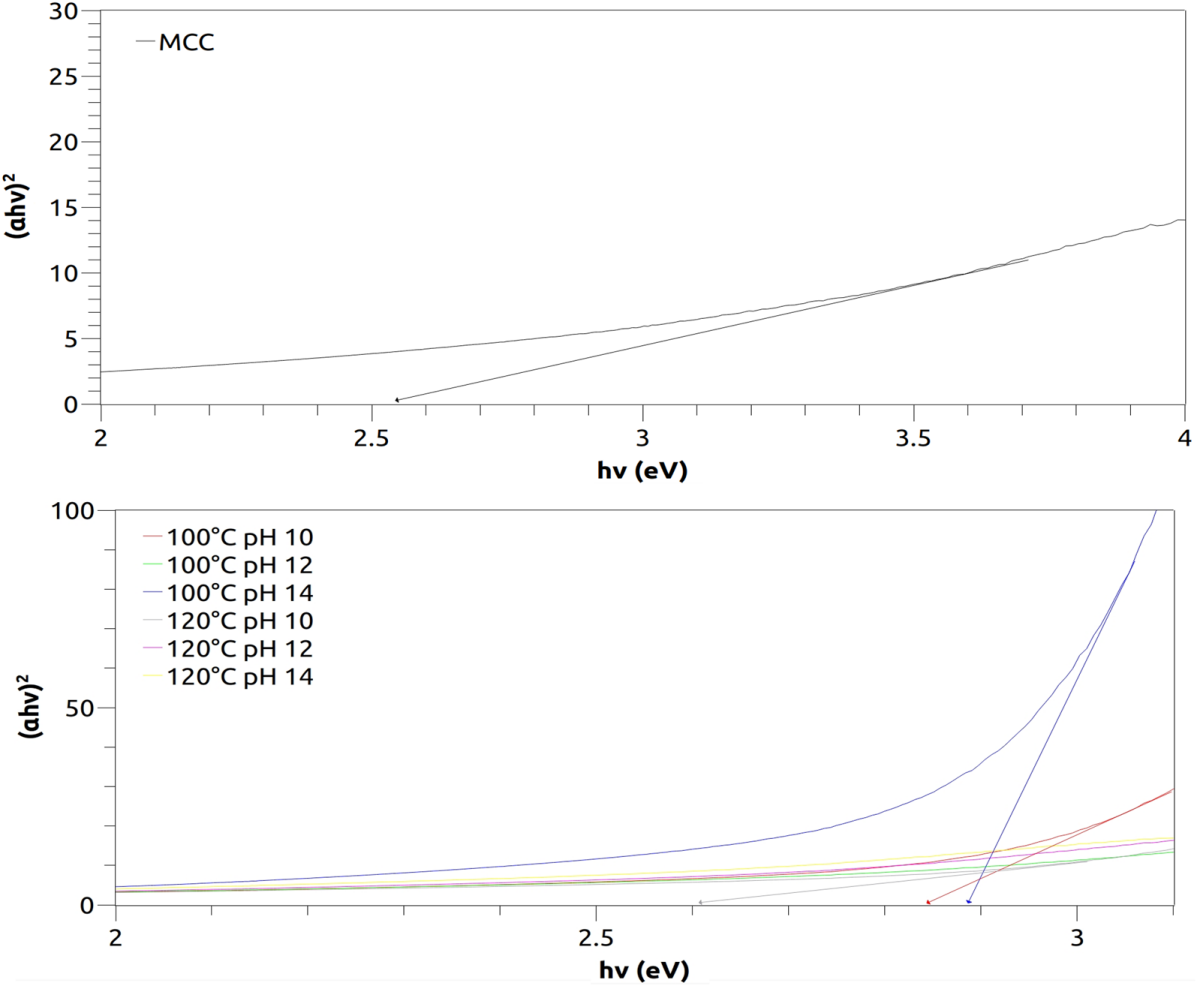
UV–Vis spectra of the microcrystalline cellulose (upper) and the hybrid nanostructures composed of CeO_2_@MCC (lower).

Figure 4 illustrates the UV–Vis spectrum of the as-prepared samples synthesized at 100 °C and 120 °C for 8 min by the MAH method. The optical band gap energy (E_gap_) was calculated according to the method proposed by Kubelka and Munk, already described elsewhere^34^⁠. This methodology is based on the transformation of diffuse reflectance measurements to estimate E_gap_ values with good accuracy within the limits of assumptions when modeled in three dimensions^71^⁠.

Despite the theoretical value of 6 eV for mono-crystalline ceria^72^⁠, the experimental E_gap_ values range from 2.5 to 3 eV, with the patterns suggesting a distribution of localized energetic levels originated from defective species, such as oxygen vacancies and Ce(III) atoms, essential in the scavenging of reactive oxygen species (ROS).

The values obtained were lower than those reported in the literature. For instance, Phokha et al.^73^⁠ calculated direct band gap values ranging from 3.00 to 3.10 eV for monodisperse CeO_2_ nanospheres synthesized by PVP-assisted hydrothermal method, while Palard et al.^74^⁠ reported estimated band gaps of 3.56–3.76 eV for undoped and doped cerium oxide nanoparticles with the following chemical formula: Ce_1−x_M_x_O_2−(x/2)_ (M = Y or Gd and x = 0 or 0.15) by different co-precipitation protocols at room temperature. We also found studies suggesting that the morphology of CeO_2_ nanostructures can influence the band gap^61,65^, with distinct undoped CeO_2_ nanostructures showing different *E*_*gap*_ values depending on their exposed surface.

In terms of COVID-19-related systemic complications, the homeostasis between pro-oxidants and antioxidants is disturbed due to redox imbalance, which results in cellular damage, making the disease more intense. As a potent reactive oxygen species (ROS) scavenger, nanoceria can be a potential tool for COVID-19 pathological damage reduction^75^⁠.

In this way, we can conclude that the distinct synthesis conditions (pH, temperature, and consequently pressure) reached during microwave irradiation are responsible for the creation of intrinsic defects that support the multi-functionality of the studied hybrid ceria nanostructures^76^⁠ since their structural, morphological, photoluminescent, magnetic and electric properties are strongly correlated with the quantum electronic transitions between rare-earth f-states found in the defective species.

### Fourier-transform infrared (FT-IR) spectroscopy

**Figure 5:**
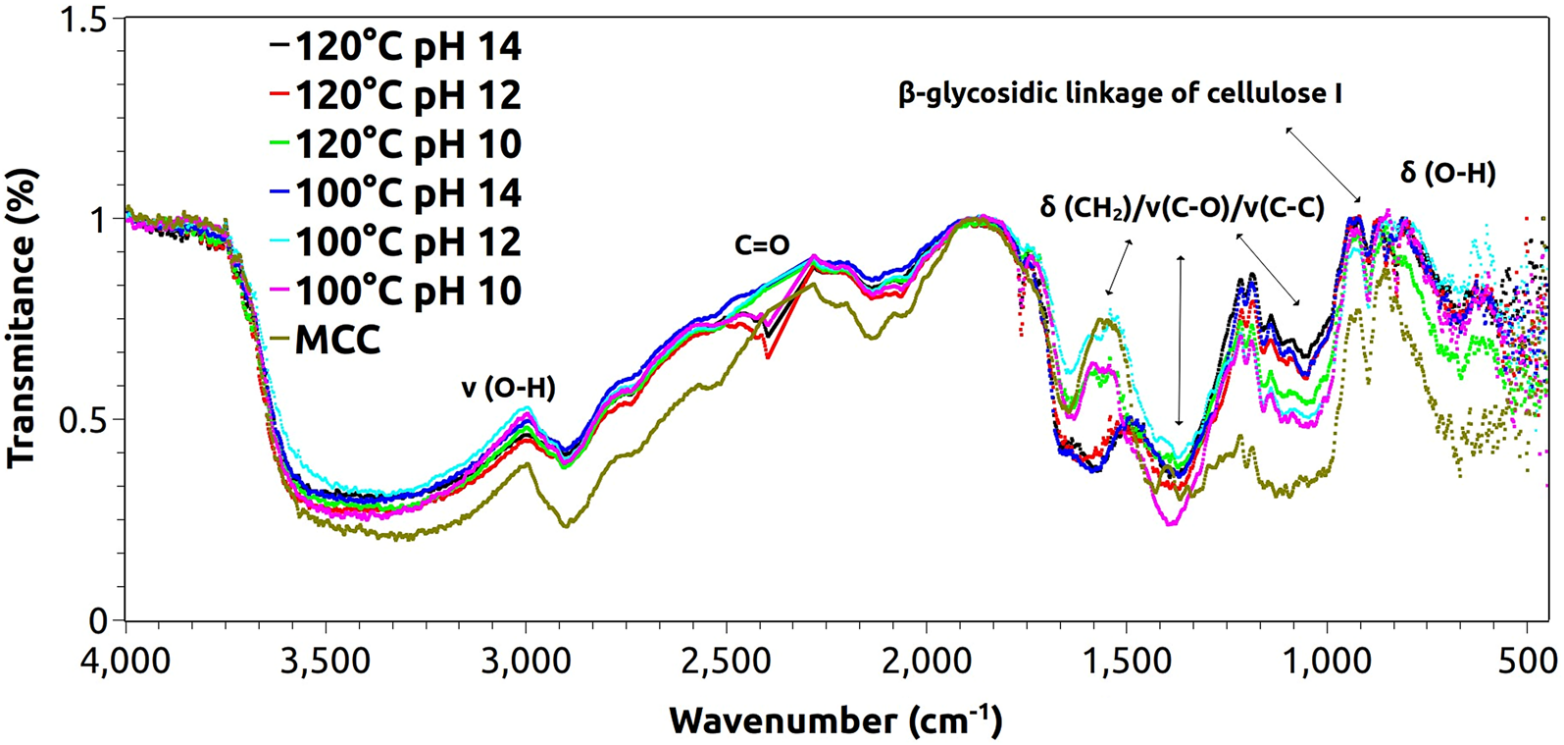
FT-IR spectra of the as-synthesized particles and the raw microcrystalline cellulose.

The FT-IR spectra of the as-synthesized samples are shown in Fig. 5. The results depict strong bands around 3500, 2300, 1500 and 1050 cm^−1^ corresponding to stretching and deflection modes of hydroxyl (O–H) and carbonyl (C=O) radicals^77^⁠. The same modes near 1408 and 1600 cm^−1^ were found by Yulizar et al*.*^78^⁠, attributing them to C=O (carbonyl) and C–C aromatic groups.

The modes below 1000 cm^−1^ indicate the presence of β- glycosidic linkage from the cellulose backbone, while the event close to 500 cm^−1^ corresponds to stretching/deflection modes of cerium oxide octahedral [CeO_8_] clusters in a fluorite-type crystalline structure^79^⁠.

The FT-IR bands around 1500–900 cm^−1^ are similar to those found in literature for commercial CeO_2_ powders^74^⁠ and CeO_2_ nanoparticles^80^⁠, and corroborates the formation of the nanoparticles by the MAH route.

### Raman spectroscopy

**Figure 6:**
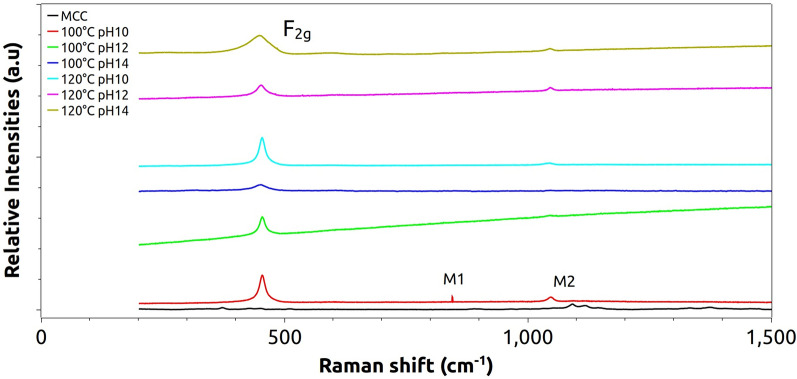
Raman spectra of the hybrid structures and the MCC.

Figure 6 shows the presence of the typical F_2g_ symmetric vibrational mode of the O atoms around each cation at 450 cm^−1^, associated with the cubic fluorite structure of ceria^81^⁠. The symmetric breathing mode near 450 cm^−1^ is nearly independent of the cation mass because only the O atoms move^82^⁠. According to previous publications, Cu/CeO_2_-hollow nanospheres obtained by a hydrothermal method also depicted the F_2g_ mode close to 458 cm^−1^, while another mode about 590 cm^−1^ was assigned to defect-induced (DI) mode^83^⁠, bounded with oxygen vacancies owing to the existence of Ce^3+^.

The second-order M1 mode around 850 cm^−1^, clearly seen in the system synthesized at 100 °C and pH 10, reveals the reduction of Ce^4+^ to Ce^3+^, according to literature^84^, suggesting an alteration of the short-range order symmetry and an increase in the concentration of oxygen-related defects within the structure of the material^85^⁠, while the M2 mode around 1050 cm^−1^ is attributed to oxygen vacancies intrinsically created as follows (Eq. 1):

1 $$ {\text{O}}_{{\text{o}}}^{{\text{x}}} + {\text{ Ce}}_{{{\text{Ce}}}}^{{\text{x}}} \to {\text{ V}}_{{{\text{O}}^{ \cdot \cdot } }} + {\text{ 2Ce}}_{{{\text{Ce}}}}^{^{\prime}} + \, \raise.5ex\hbox{$\scriptstyle 1$}\kern-.1em/ \kern-.15em\lower.25ex\hbox{$\scriptstyle 2$} {\text{ O}}_{{2}} $$

where O_o_^x^ and Ce_Ce_^x^ represent oxygen and cerium atoms at their regular sites, V_O_^..^ represents a doubly ionized oxygen vacancy and Ce_Ce_^’^ corresponds to the substitution of Ce(III) by Ce(IV) with effective charge −1^86^⁠.

The broadening of the F_2g_ mode for higher temperature and pH values can be associated with the expansion of the octahedral [CeO_8_] sites, which occurs when Ce(III) replaces Ce(IV) during oxygen vacancy creation^87^⁠, thus decreasing the short-range symmetry after temperature rise^77^⁠. This behavior is clearly depicted for the sample synthesized at 100 °C, pH 10.

In terms of MCC response, its general aspect is very close to other polysaccharides with stretching of (C–O) and (C–C) groups and deformation of (CH_2_) radicals within 800–1500 cm^−1^. Besides, modes at 380–460 cm^−1^ can be assigned to vibration/deformation of (CCC), (CO) and (CCO) rings^88^⁠.

It is also possible to estimate the average size of the CeO_2_ clusters by the following equation^89^⁠:

2 $$ \Gamma {-} \, 10\;{\text{cm}}^{ - 1} = \, 124.7/d $$

where Г, expressed in cm^−1^, is the FWHM parameter of the active F_2g_ mode regarding Ce–O vibration, and *d* is the average crystallite size in nm.

It can be seen that the *d* values are close to those estimated by XRD results (*D*), corroborating the presence of quantum dots grown along the cellulose surface. Additionally, the pH is found to influence more than the temperature since the average sizes reduce as the temperature increases, demonstrating the role of the microcrystalline cellulose as a template, which ends up preventing the growth of ceria quantum dots.

It is worth mentioning that intrinsic generation of oxygen vacancies can occur within the ceria structure. To understand that, it is necessary to consider the resonant equilibrium between the ordered and disordered [CeO_8_] clusters^90^⁠, represented according to the Kroger-Vink notation^91,92^:

3 $$ \left[ {{\text{CeO}}_{{8}} } \right]_{{\text{o}}}^{{\text{x}}} \leftrightarrow \, \left[ {{\text{CeO}}_{{8}} } \right]_{{\text{d}}}^{{\text{x}}} $$

Then, the following situations can arise:

4 $$ \left[ {{\text{CeO}}_{{\text{8}}} } \right]_{{\text{o}}} ^{{\text{x}}}  + {\text{ }}\left[ {{\text{CeO}}_{{\text{8}}} } \right]_{{\text{d}}} ^{{\text{x}}}  \to {\text{ }}\left[ {{\text{CeO}}_{{\text{8}}} } \right]_{{\text{o}}} ^{\prime }  + {\text{ }}\left[ {{\text{CeO}}_{{\text{7}}}  \cdot {\text{V}}_{{{\text{O}}^{ \cdot } }} } \right]{\text{ }} + {\text{ 1}}/{\text{2O}}_{{\text{2}}}  $$

5 $$ \left[ {{\text{CeO}}_{{\text{8}}} } \right]_{{\text{o}}} ^{{\text{x}}}  + {\text{ }}\left[ {{\text{CeO}}_{{\text{7}}}  \cdot {\text{V}}_{{{\text{O}}^{ \cdot } }} } \right]{\text{ }} \to {\text{ }}\left[ {{\text{CeO}}_{{\text{8}}} } \right]_{{\text{o}}} ^{\prime }  + {\text{ }}\left[ {{\text{CeO}}_{{\text{7}}}  \cdot {\text{V}}_{{{\text{O}}^{{ \cdot  \cdot }} }} } \right] $$

6 $$ \left[ {{\text{CeO}}_{{\text{8}}} } \right]_{{\text{o}}} ^{{\text{x}}}  + {\text{ }}\left[ {{\text{CeO}}_{{\text{7}}}  \cdot {\text{V}}_{{\text{O}}} ^{{\text{x}}} } \right]{\text{ }} \to {\text{ }}\left[ {{\text{CeO}}_{{\text{8}}} } \right]_{{\text{o}}} ^{\prime }  + {\text{ }}\left[ {{\text{CeO}}_{{\text{7}}}  \cdot {\text{V}}_{{{\text{O}}^{ \cdot } }} } \right] $$

7 $$ \left[ {{\text{CeO}}_{{\text{7}}}  \cdot {\text{V}}_{{\text{O}}} ^{{\text{x}}} } \right]{\text{ }} + {\text{ }}\left[ {{\text{CeO}}_{{\text{7}}}  \cdot {\text{V}}_{{{\text{O}}^{ \cdot } }} } \right]{\text{ }} \to {\text{ }}\left[ {{\text{CeO}}_{{\text{7}}}  \cdot {\text{V}}_{{\text{O}}} ^{{\text{x}}} } \right]_{{\text{o}}} ^{\prime }  + {\text{ }}\left[ {{\text{CeO}}_{{\text{7}}}  \cdot {\text{V}}_{{{\text{O}}^{{ \cdot  \cdot }} }} } \right] $$

where Ce represents the rare-earth element, O is the oxygen atoms and V_o_ is the oxygen vacancies, which can be neutral (V_o_^x^), singly (V_o_^.^) or doubly ionized (V_o_^..^). The [CeO_8_]^’^-type clusters with a negative charge represents the Ce^3+^ species.

### Positron annihilation lifetime spectroscopy: defect structure

When measuring different crystalline materials using PALS, in general from spectra decomposition, several lifetime components can be obtained; each of them is characterized by a lifetime τ_i_ and an associated intensity I_i_. The state i can be the delocalized one in the lattice (bulk state) or localized states at different defect sites where positrons become trapped and annihilated. In the last case, the lifetime value reflects the size of the open volume associated with the defect in which the positrons are annihilated. Therefore, an increase in the lifetime means that positrons are annihilating into bigger open volumes. Furthermore, their associated intensities I_i_ provide information about the defect concentrations. Correspondingly, an increase in the I_i_ leads to an increase in the defect concentration. In polymeric samples, a long-lived lifetime component (> 1.5 ns) is revealed, commonly associated with positronium (Ps) annihilation in this kind of materials, and more specifically with the ortho-Ps triplet state (τ_o-Ps_, I_o-Ps_)^93^⁠. By using a simple semi-empirical quantum–mechanical model of Tao-Eldrup^94,95^, it is possible to directly determine the size and relative concentration of the average free nanohole volume from the o-Ps lifetime.

From the measurements of the samples studied in this work, all PALS spectra could be satisfactorily decomposed using three discrete lifetime components, which are presented in Table 3.

First of all, the raw MCC sample was measured. From the analysis of the results, a non-negligible long-lived τ_3_ component of 1670 ps typical for polymeric systems was obtained. Through the Tao-Eldrup model^94,95^ it was possible to calculate the average nanohole size (0.253 ± 0.003 nm), which is in very good agreement with the one and only value reported in the literature for cellulose nanocrystal films from PALS measurements^96^⁠.

Regarding the results obtained from the measurements of CeO_2_@MCC samples listed in Table 3, a dominant second lifetime component τ_2_ with lifetimes between 353 and 373 ps and associated intensities between 53 and 67% can be observed. The shortest lifetime component τ_1_ has values ranging from 212 to 223 ps with associated intensities varying from 30 to 44%.

For the samples synthesized at 100 °C, the τ_1_ values systematically decrease as the pH increases, ranging from ~ 216 ps (pH 10) to ~ 202 ps (pH 14). In addition, the associated intensities decrease from 42 (pH 10) to 30% (pH 14). At the same time, for all samples the τ_2_ values remain almost constant within the experimental scatter (τ_2_ ~ 362 ps), while the associated intensities increase from 56 (pH 10) to 67% (pH = 14).

The rise of the synthesis temperature to 120 °C induces an increase in the τ_1_ values when compared to those obtained for the samples synthesized at 100 °C. It is noteworthy that again the τ_1_ values systematically decrease as the pH increases, varying from ~ 223 ps (pH 10) to ~ 208 ps (pH 14). A similar behavior to that observed for the CeO_2_@MCC samples synthesized at 100 °C occurs in the intensity I_1_, that is, I_1_ varies from 44 (pH 10) and 32% (pH 14). The second lifetime component τ_2_ decreases from ~ 373 ps for the smaller pH value to ~ 353 ps for the higher one, while their associated intensities increase from 53% (pH 10) to 66% (pH 14).

In all samples, it can be observed in the table that the intensity associated with the long-lived lifetime is small (I_3_ < 3%). This result reveals that almost all positrons are annihilating in the CeO_2_ QDs, while an almost negligible number of positrons would form o-Ps annihilating in open volumes present in the MCC polymeric matrix, which corroborates the role of the cellulose as a template for the nucleation and growth of the ceria particles. As the aim of the present work is to study the defect structure of the CeO_2_@MCC samples, this lifetime component does not bring any useful information. Therefore, we consider that the CeO_2_ particles act as highly effective positron traps, meaning that positrons are localized within the CeO_2_ QDs where they annihilate.

In this case, the real information about the defect structure is condensed in the first and second lifetime components. Similar interpretation about the assignment of the different lifetime components was also reported by different authors^42,44,97,98^.

When compared to the typical positron lifetimes for monovacancies in solids reported in the literature, the high values obtained for the second lifetime components in all samples can be assigned to positron annihilated in traps associated with open volumes resembling small vacancy clusters^97–99^. In particular, from the study of changes in the defect structure of different CeO_2_-based nanostructured systems due to variations in the synthesis process using PALS, several authors reported the presence of a dominant positron lifetime τ_2_ with values ranging from 300 to 450 ps (see Table 3). Additionally, these authors found a shorter lifetime τ_1_ with values between 236 and 262 ps^97,98^. In these papers, the second lifetime component was attributed to positron annihilation in large surface oxygen vacancy clusters (OVCs), while the shorter positron lifetime was assigned to positrons annihilated in small neutral Ce^3+^-oxygen vacancy associations, demonstrating the presence of fundamental species for virucidal capability.

On the other hand, the results obtained from the study of the (111) surface of pure CeO_2_ using dynamic force microscopy (DFM) and scanning tunneling microscopy (STM) as well as DFT calculations indicated that oxygen vacancies tend to form compact clusters in the surface or sub-surface of the CeO_2_ samples with different morphologies, such as triangular clusters, extended lines and chains^100,101^. Furthermore, DFT results revealed that these OVCs are exclusively coordinated by Ce^3+^ ions^100^⁠.

Based on the experimental and theoretical findings described above, the values of the characteristic parameters of the second lifetime component presented in Table 3 indicate abundant trapping and annihilation of positrons at OVCs located at the CeO_2_ QD surfaces.

As it can be seen in Table 3, for the lowest synthesis temperature the τ_2_ values are almost independent of the pH reaction. Therefore, it can be concluded that in this set of samples the sizes of OVCs are practically constant. In the case of samples synthesized at 120 °C, τ_2_ slightly decreases when pH increases, with the corresponding OVC sizes following the same behavior.

In contrast, taking into account the mean crystallite size (D) obtained from XRD and Raman measurements (*D* values in Table 1 and *d* values in Table 2, respectively) and the values of intensity associated with the second positron lifetime component characterizing the oxygen vacancy clusters (Table 3), it can be seen that for each sample there is a direct correlation between these parameters (D and I_2_). In fact, for all cases a reduction in the mean crystallite size is correlated with an increase in I_2_. From these behaviors, it could be concluded that the OVCs are located at the surface of the CeO_2_ particles. It means that smaller particles have higher specific surface areas, which would enable the formation of more oxygen vacancy clusters.

**Table 2:** Crystallite sizes from Raman spectroscopy.

Temperature (°C)	pH	Г (cm^−1^)	*d* (nm)
100	10	20.65	11.70
	12	25.71	7.93
	14	48.26	3.25
120	10	20.84	11.50
	12	33.98	5.20
	14	42.98	3.78

**Table 3:** Characteristic positron lifetimes and associated intensities obtained from the decomposition of PALS spectra for the raw MCC sample and the CeO_2_@MCC samples under different synthesis parameters.

T(°C)	pH	τ_1_ (ps)	I_1_ (%)	τ_2_ (ps)	I_2_ (%)	τ_3_ (ps)	I_3_ (%)
**Raw MCC**							
–	–	208 ± 1	38 ± 1	400 ± 1	52 ± 1	1670 ± 10	10 ± 1
**CeO**_**2**_**@MCC**							
100	10	216 ± 2	42 ± 1	363 ± 4	56 ± 1	1520 ± 10	2 ± 1
	12	212 ± 2	34 ± 1	360 ± 4	63 ± 1	1500 ± 10	3 ± 1
	14	202 ± 2	30 ± 1	362 ± 4	67 ± 1	1500 ± 10	3 ± 1
120	10	223 ± 3	44 ± 1	373 ± 5	53 ± 1	1540 ± 10	3 ± 1
	12	217 ± 2	37 ± 1	360 ± 4	60 ± 1	1570 ± 10	3 ± 1
	14	208 ± 2	32 ± 1	353 ± 3	66 ± 1	1470 ± 10	2 ± 1

Regarding the first positron lifetime values measured in the present work, they are systematically higher than the typical one reported in the literature for positrons annihilated in defect-free pure CeO_2_, *i.e.*, $\uptau$_b_ = 187 ps^98^⁠. Consequently, our τ_1_ values can be interpreted in terms of positron annihilation in much smaller open volume defects than the OVCs, with a typical size equivalent to a mono- and/or di-vacancy.

In the literature, it has been demonstrated that oxygen vacancies (V_O_) are the most abundant defects in pure CeO_2_^102^, however, it is also well known that isolated V_O_ are not effective trapping centers for positrons because they are positively charged^103^⁠. Therefore, the behavior of $\uptau$_1_ reported herein cannot be only explained in terms of positron annihilation in isolated V_O_. In such a case, the defects acting as a positron trap should be a more complex vacancy-like defect such as a V_O_ associated with a negative ion. In studies on pure CeO_2_, it has been shown that defects characterized by lifetimes between 236 and 262 ps correspond to positrons annihilated in small neutral Ce^3+^–V_O_ associates^97,98^⁠.

As presented in Table 3, the τ_1_ values measured for all CeO_2_@MCC samples are higher than the corresponding lifetime values found in the literature for defect-free ceria, but smaller than those reported for Ce^3+^–V_O_ associates. Accordingly, our $\uptau$_1_ values reveal that positrons annihilate in a mixed state containing both defect-free ceria and Ce^3+^–V_O_ associates. Additionally, these $\uptau$_1_ values systematically decrease as the defect concentration (cerium vacancy-like associates) decreases.

**Figure 7:**
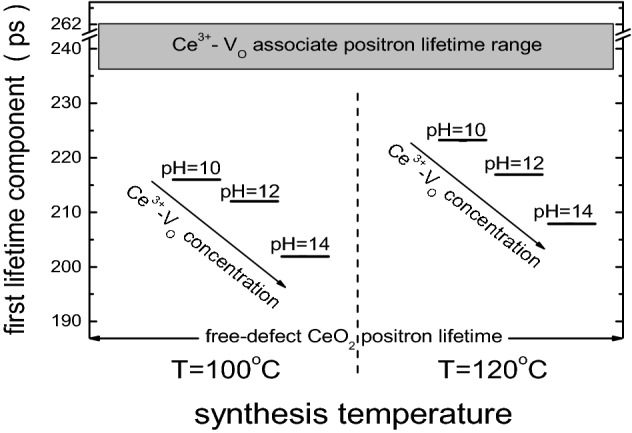
Schematic representation of the τ_1_ evolution as a function of reaction pH for the different synthesis temperatures. The corresponding lifetimes for the free-defect CeO_2_ and the Ce^3+^-oxygen vacancy associates are also presented.

Finally, from the values presented in Table 3 it can be concluded that an increase in the synthesis temperature induces an increment in the concentration of Ce-vacancy associates. Besides, for a fixed synthesis temperature our results indicate that the concentration of Ce^3+^–V_O_ associates is dependent on the reaction pH, that is, the higher the pH, the lower the concentration of these defects. This behavior is schematized in Fig. 7.

Therefore, the main PALS results can be summarized as follows:

- Positrons are strongly attracted to the CeO_2_ QDs, revealing the presence of two different kinds of oxygen defects: (1) oxygen vacancy clusters located at the surface of the particles, and (2) Ce^3+^-oxygen vacancy associates located either at the surface or inside the NP, which corroborates the presence of fundamental species required for virucidal capability.
- An increase of the synthesis temperature induces an increment in the concentration of Ce^3+^-oxygen vacancy associates; and for a given synthesis temperature, an increment in the pH reaction induces a reduction in the NP sizes and in the concentration of cerium vacancy associates.

Positron results showed that both temperature and pH influence the concentration of ceria defects along the cellulose surface. The application of the semiconductor for biocidal activity is directly related to the defect density (in this case the Ce(III)-oxygen vacancy associates) due to the formation of clusters with lower charge density. Thus, in the crystal structure of ceria there is the formation of dipoles with charge transfer to the prohibited region of the ceria band gap as a result of the increase in polarized electrons. In ordered clusters there is an increase in charge density, whereas in disordered ones the charge density decreases. This charge imbalance facilitates charge transfer and absorption to oxygen and water molecules as well as ROS formation, according to Eqs. (8–13).

8 $$ \left[ {{\text{CeO}}_{{\text{8}}} } \right]_{{\text{o}}} ^{{\text{x}}}  + {\text{ }}\left[ {{\text{CeO}}_{{\text{8}}} } \right]_{{\text{d}}} ^{{\text{x}}}  \to {\text{ }}\left[ {{\text{CeO}}_{{\text{8}}} } \right]_{{\text{o}}} ^{{'~}}  + {\text{ }}\left[ {{\text{CeO}}_{{\text{8}}} } \right]_{{{\text{d}}^{ \cdot } }}  $$

9 $$ \left[ {{\text{CeO}}_{{\text{8}}} } \right]_{{{\text{d}}^{ \cdot } }}  \to {\text{ }}\left[ {{\text{CeO}}_{{\text{7}}}  \cdot {\text{V}}_{{{\text{o}}^{ \cdot } }} } \right]{\text{ }} + {\text{ 1}}/{\text{2O}}_{{\text{2}}}  $$

10 $$ \left[ {{\text{CeO}}_{{\text{8}}} } \right]_{{\text{o}}} ^{{\text{x}}}  + {\text{ }}\left[ {{\text{CeO}}_{{\text{7}}}  \cdot {\text{V}}_{{{\text{o}}^{ \cdot } }} } \right]{\text{ }} \to \left[ {{\text{CeO}}_{{\text{8}}} } \right]^{'}  + {\text{ }}\left[ {{\text{CeO}}_{{\text{7}}}  \cdot {\text{V}}_{{{\text{o}}^{{ \cdot  \cdot }} }} } \right] $$

11 $$ \left[ {{\text{CeO}}_{{8}} } \right]_{{\text{o}}}^{^{\prime}} + {\text{ O}}_{{2}} \to \, \left[ {{\text{CeO}}_{{8}} } \right]_{{\text{o}}}^{{\text{x}}} + {\text{ O}}_{{2}}^{^{\prime}} $$

12 $$ \left[ {{\text{CeO}}_{{\text{7}}}  \cdot {\text{V}}_{{{\text{o}}^{ \cdot } }} } \right]{\text{ }} + {\text{ H}}_{{\text{2}}} {\text{O }} \to {\text{ }}\left[ {{\text{CeO}}_{{\text{7}}}  \cdot {\text{V}}_{{\text{o}}} ^{{\text{x}}} } \right]{\text{ }} + {\text{ OH}}*{\text{ }} + {\text{ H}}^{ \cdot }  $$

13 $$ {\text{O}}_{{2}}^{^{\prime}} + {\text{ H}}^{ \cdot } \to {\text{ O}}_{{2}} {\text{H}}* $$

where OH* and O_2_H* are the radicals responsible for ROS formation, which will consequently oxidize bacteria, fungi and viruses.

## Conclusions

Hybrid nanostructures composed of CeO_2_@MCC were synthesized at distinct temperature and pH values by the microwave-assisted hydrothermal synthesis. Mean crystallite sizes (D) ranging between 6 and 12 nm with a decreasing D for increasing temperature was observed. A clear squared crystalline morphology, resembling a fractal of the fluorite-type cubic structure typical of pristine CeO_2_, was observed in the sample synthesized at 120 °C, pH 10. The HR-TEM images showed interplanar spacing of 0.3 nm, in accordance with the most intense peak (111) observed in the XRD. Optical band-gap values between 2.5 and 3 nm were observed in the UV–Vis results, indicating the presence of a distribution of defects, while the PALS results showed that an increase of the synthesis temperature induced an increment in the concentration of Ce^3+^-oxygen vacancy associates. XRD and PALS results indicated the formation of quantum dots along the cellulose surface. Transmission and scanning electron microscopy studies also corroborated the presence of quantum dots dispersed along the cellulose with some agglomeration due to Van Der Waals forces. Positrons were strongly attracted to the CeO_2_ QDs, revealing the presence of two different kinds of oxygen defects. These defects are essential in the scavenging of reactive oxygen species, and therefore could be used as a possible hybrid nanostructure for virus inactivation. We can then conclude that the synthesized hybrid nanoceria could be employed as a potential material for COVID-19 management.

## Supplementary Information

Supplementary Information.
