# Supplementary material for: Synthesis and defect characterization of hybrid ceria nanostructures as a possible novel therapeutic material towards COVID-19 mitigation
Source: Sci Rep. 2022 Feb 28;12:3341. doi: 10.1038/s41598-022-07200-9 (PMC8885868; doi:10.1038/s41598-022-07200-9)

SUPPLEMENTARY MATERIAL

In order to highlight the micrographs features, we performerd digital processing of the images using Photoshop. The results are seen in Figure 2 of the manuscript.

Here, we provide the original files for the sake of readers’ clarity.

Figure 2a

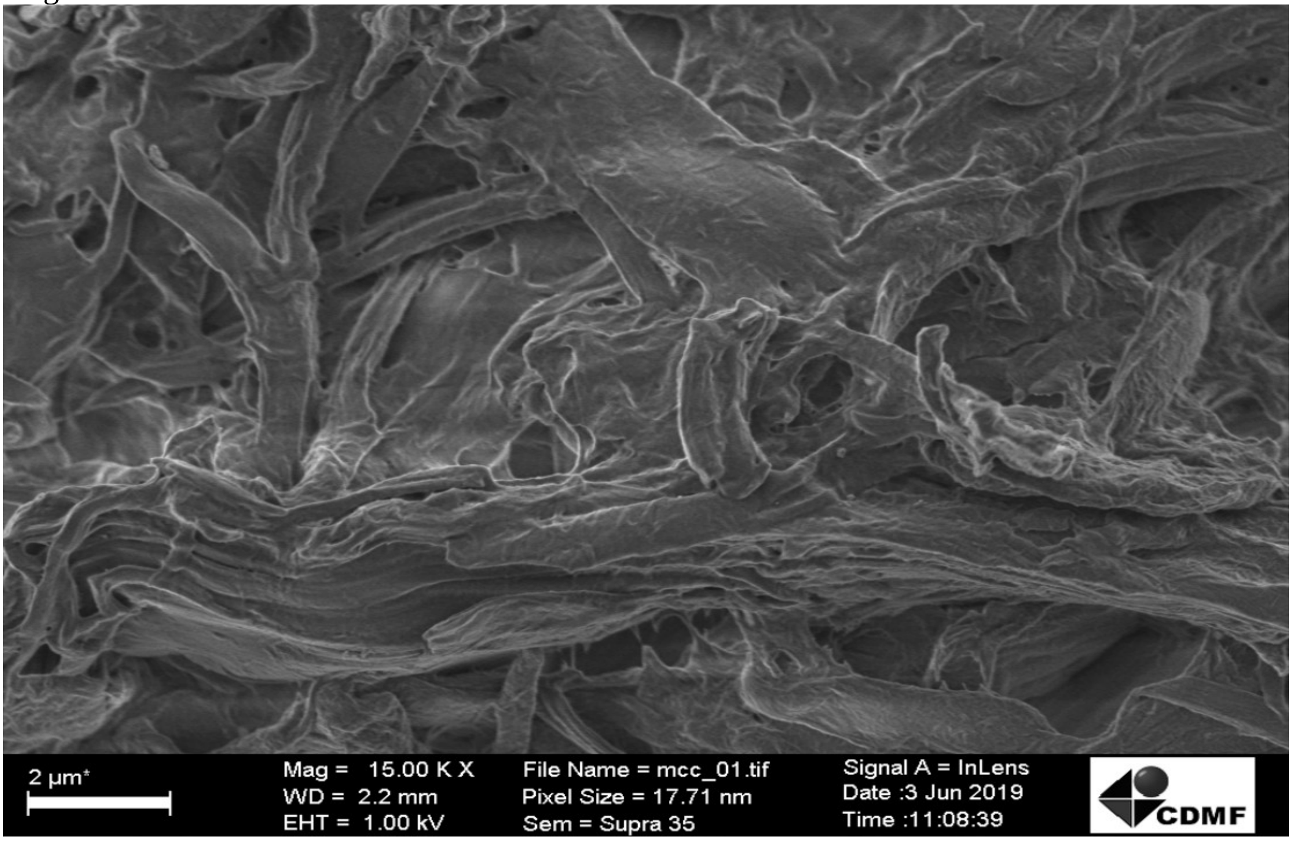

Figure 2a1

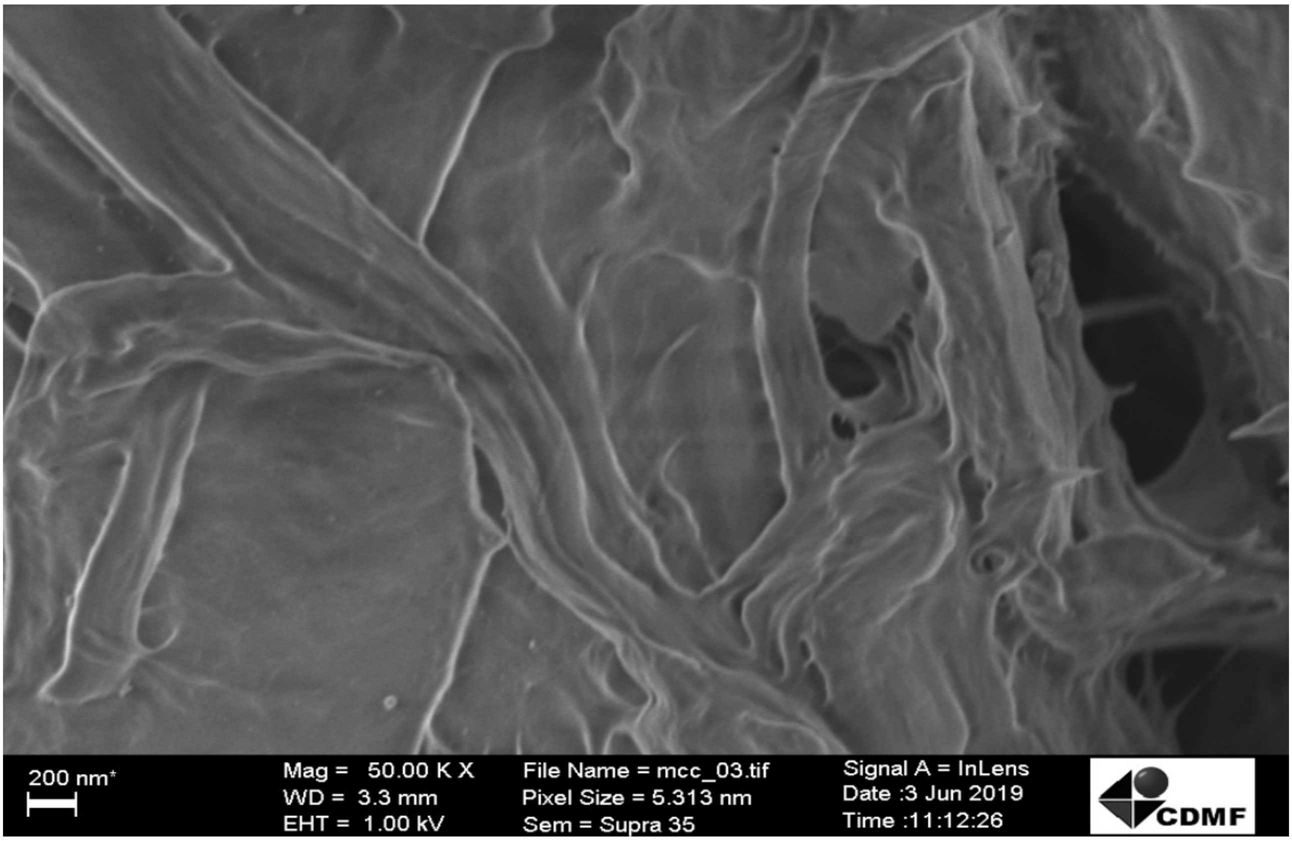

Figure 2b

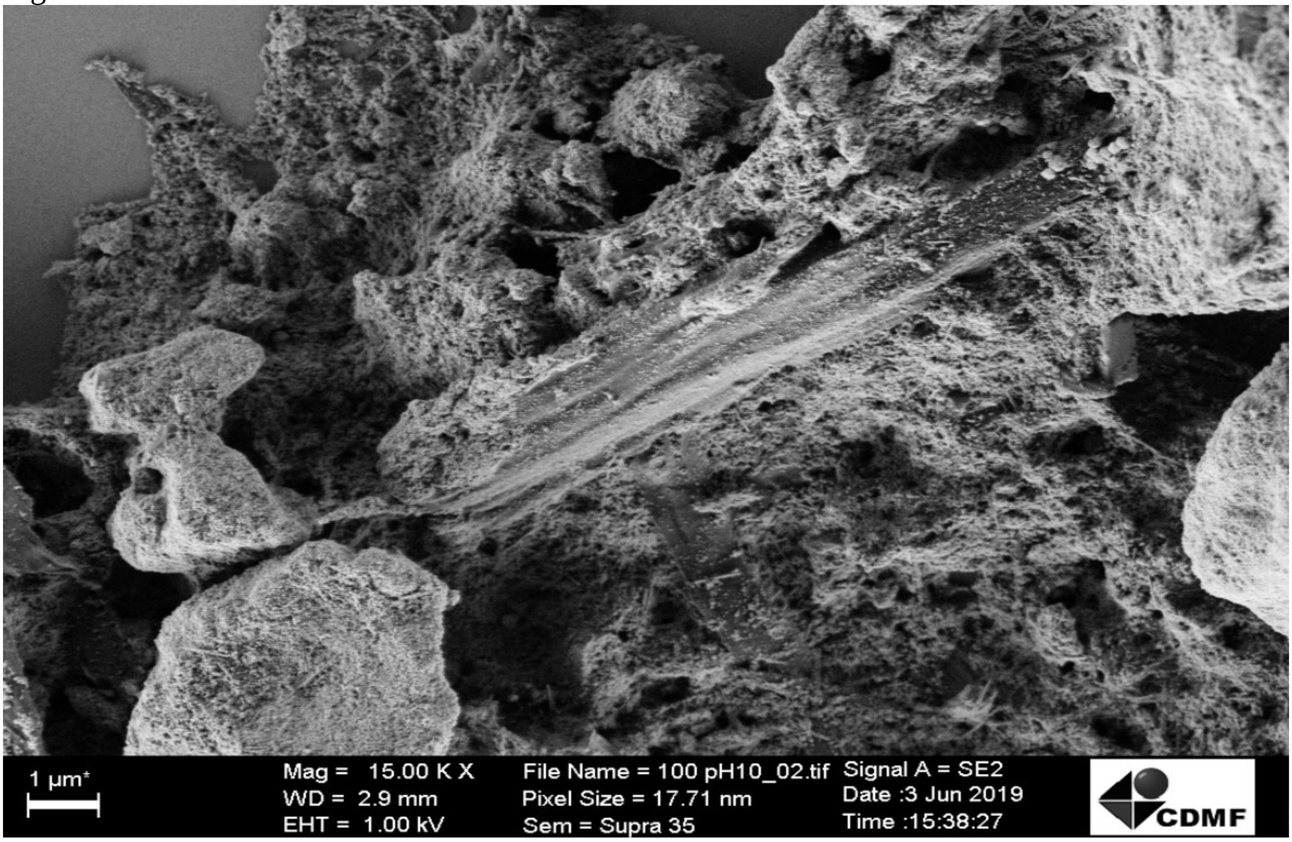

Figure 2b1

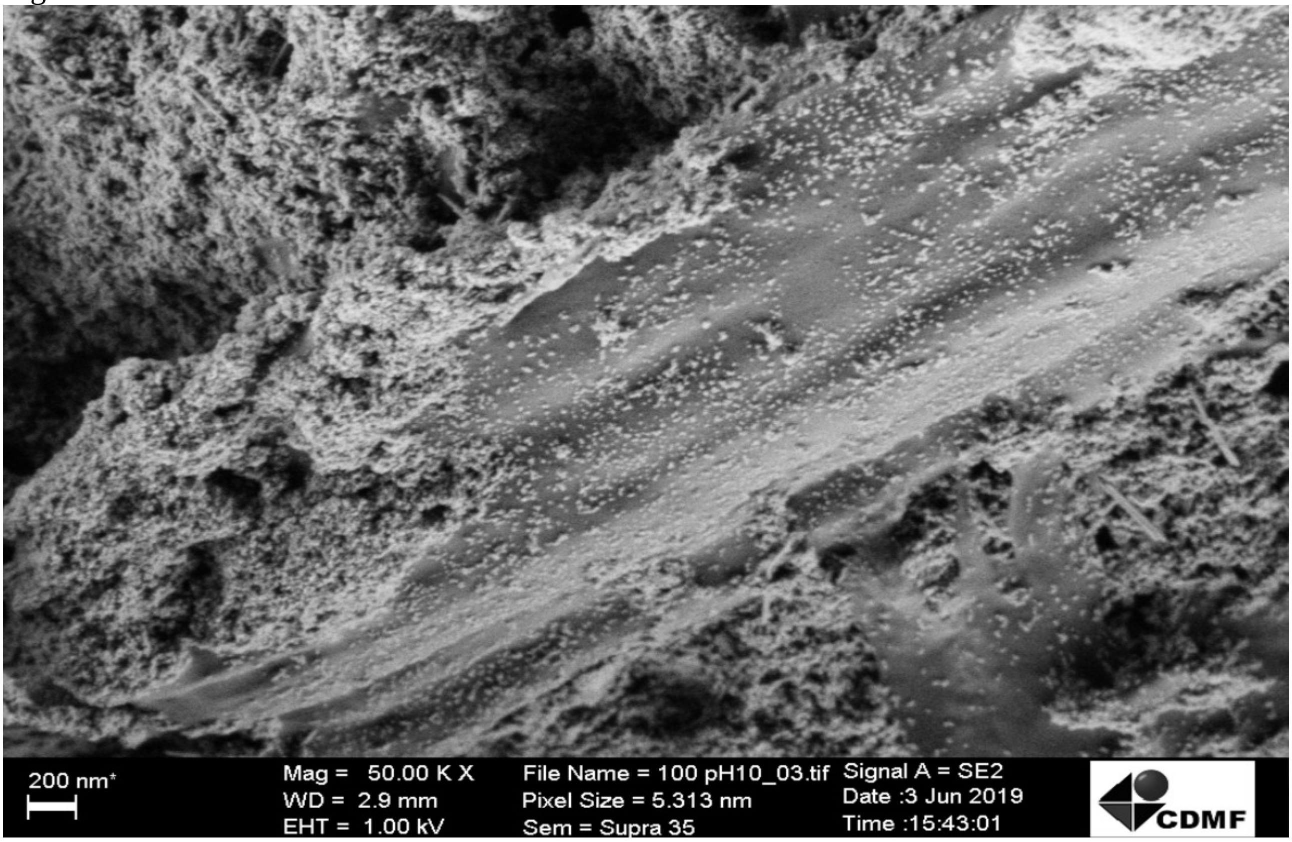

Figure 2c

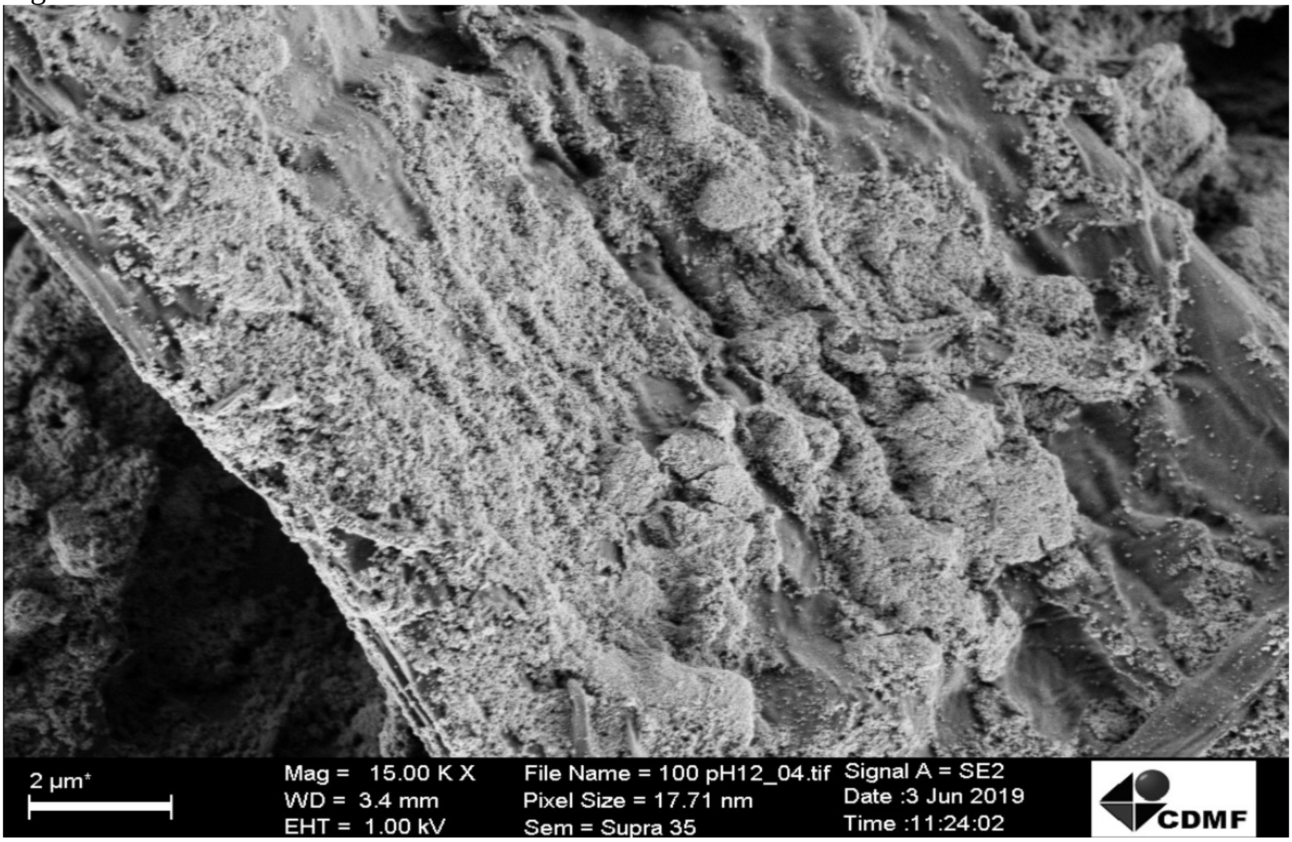

Figure 2c1

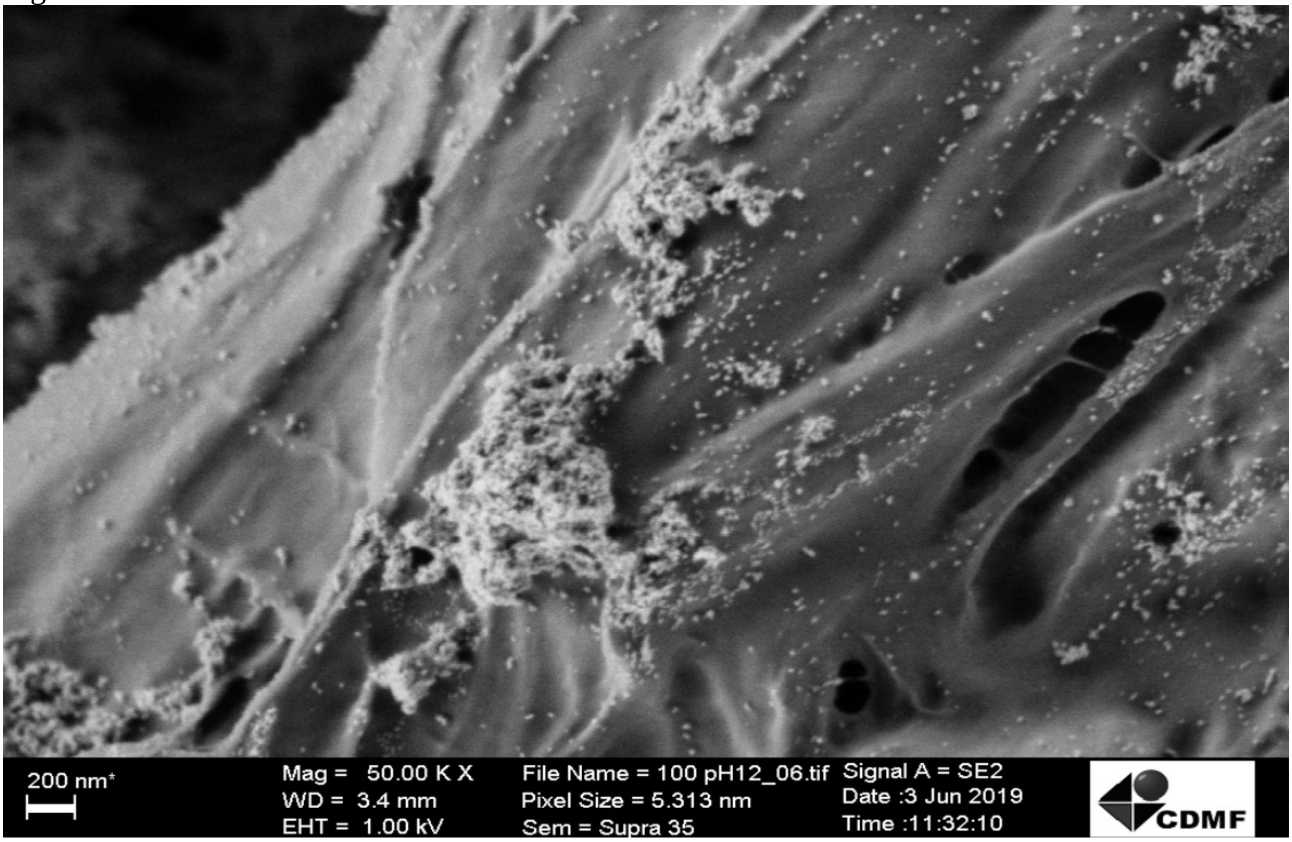

Figure 2d

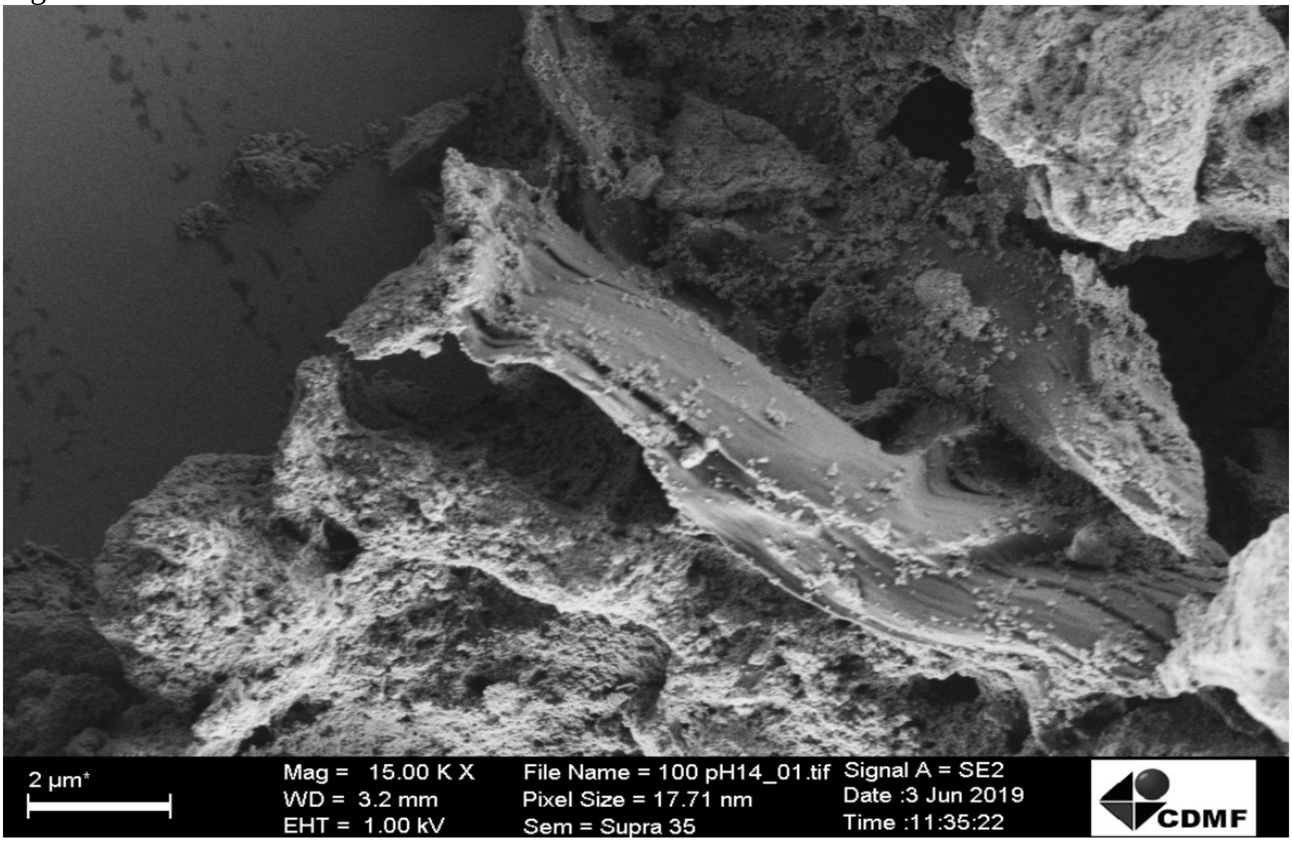

Figure 2d1

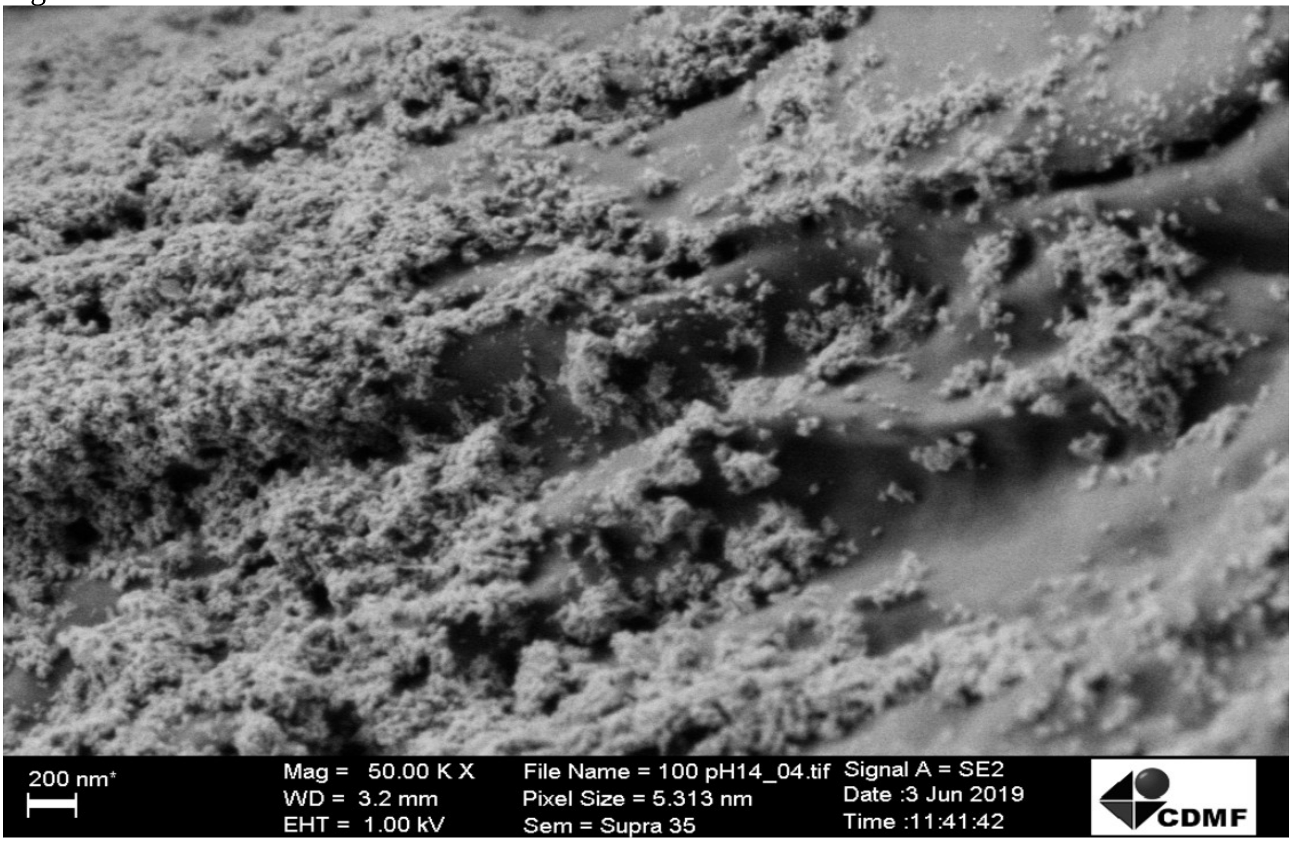

Figure 2e

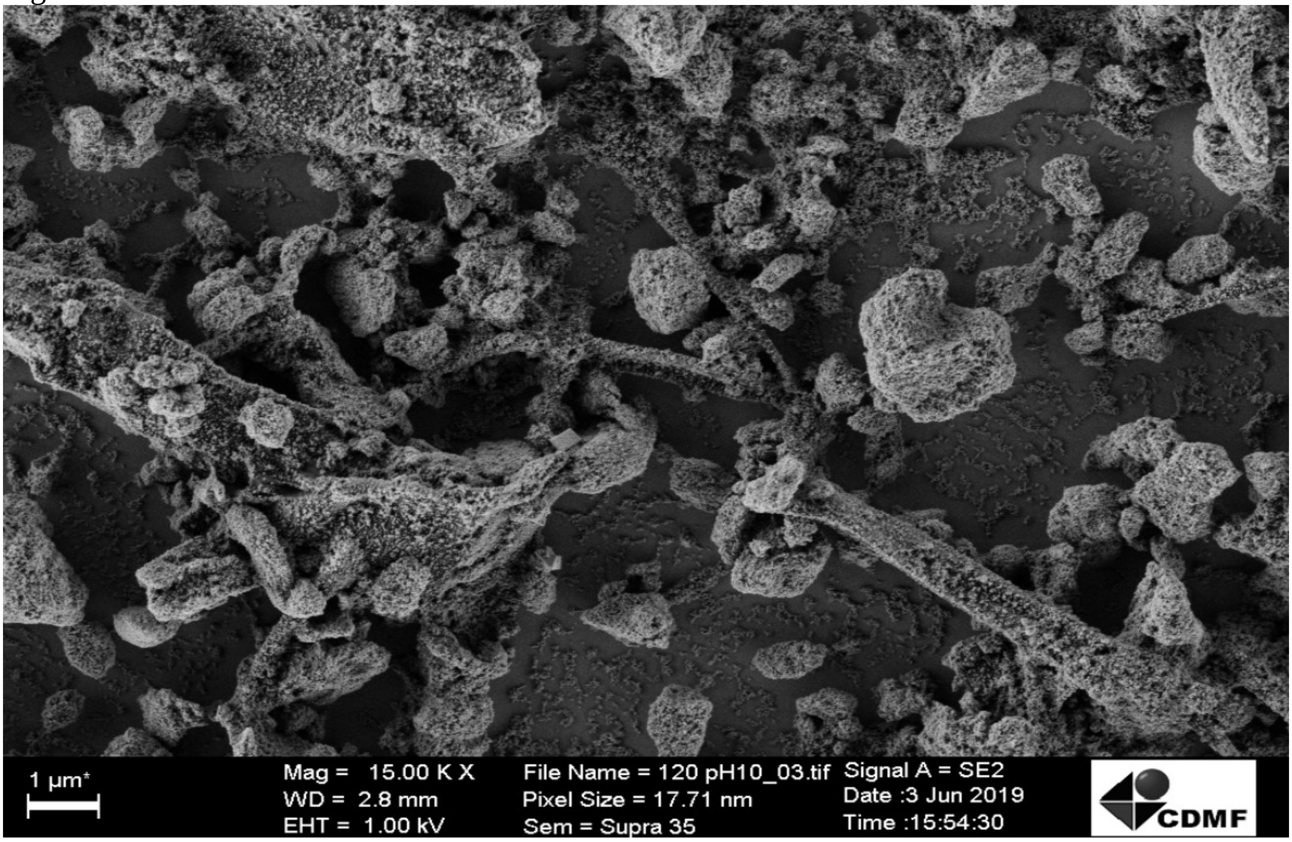

Figure 2e1

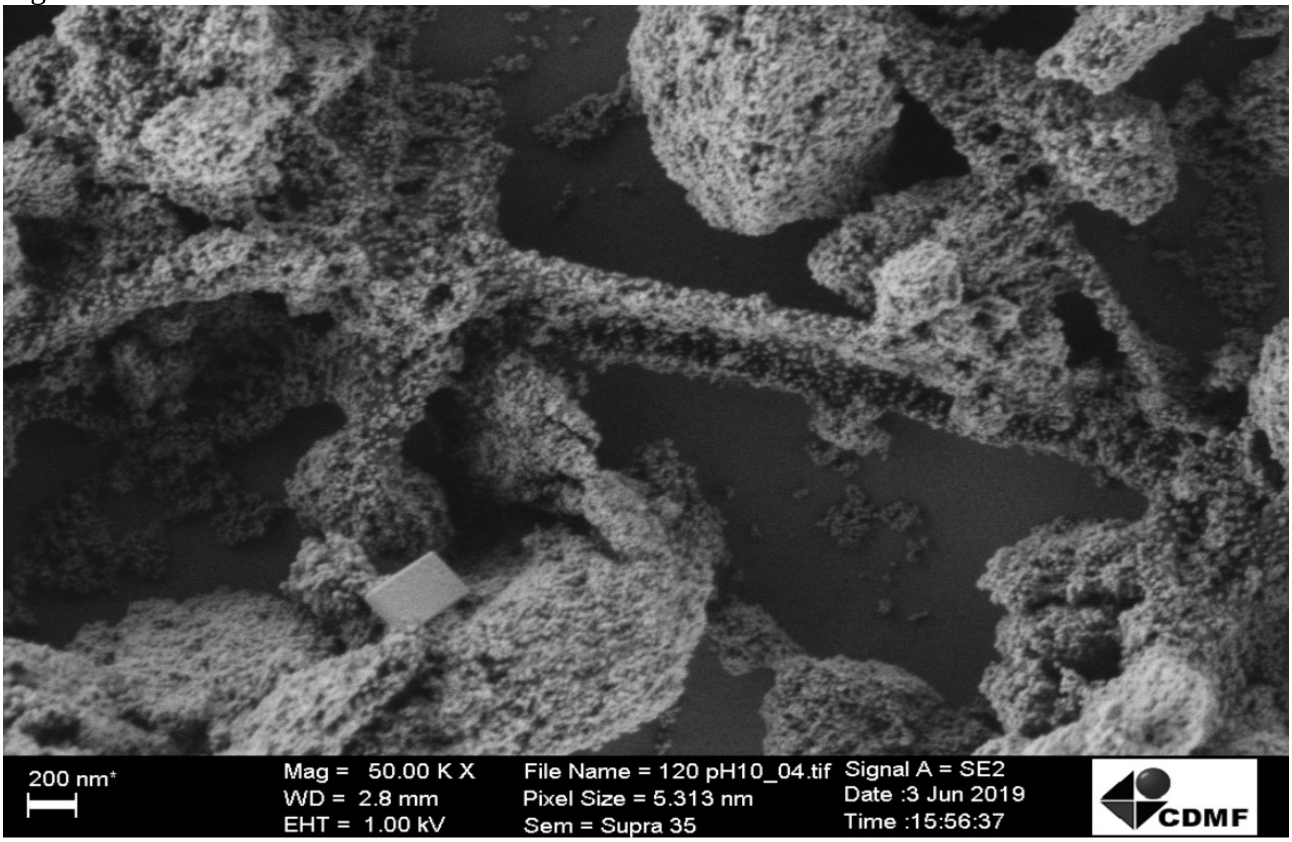

Figure 2f

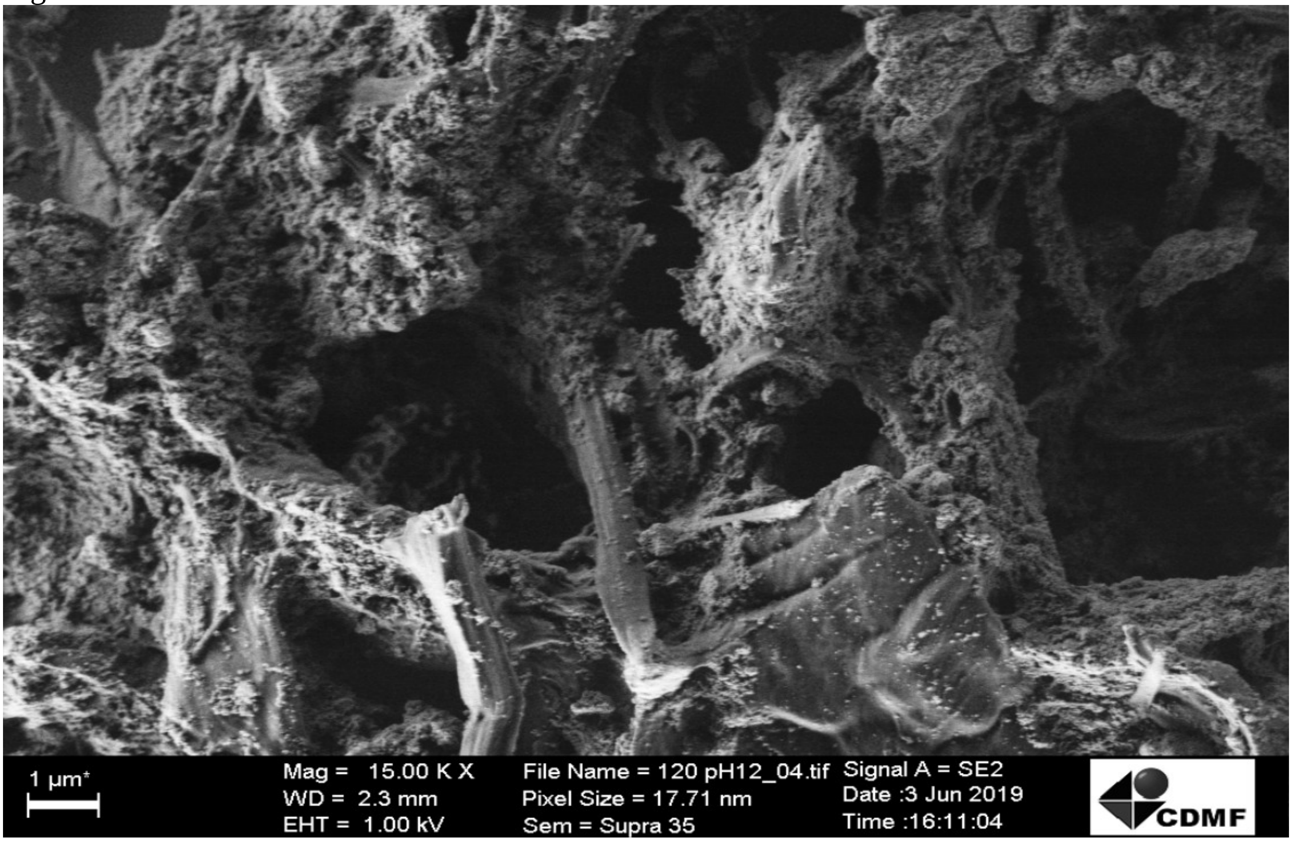

Figure 2f1

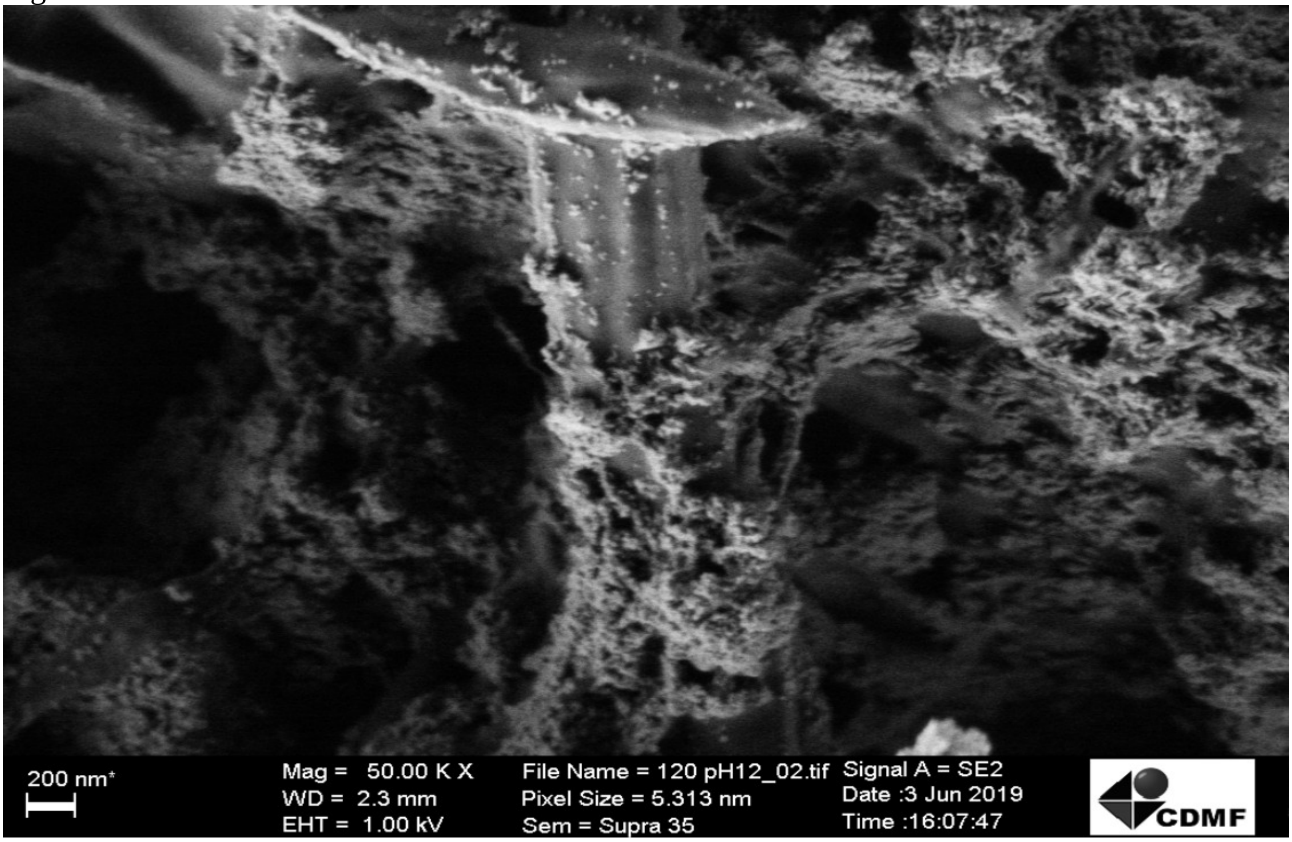

Figure 2g

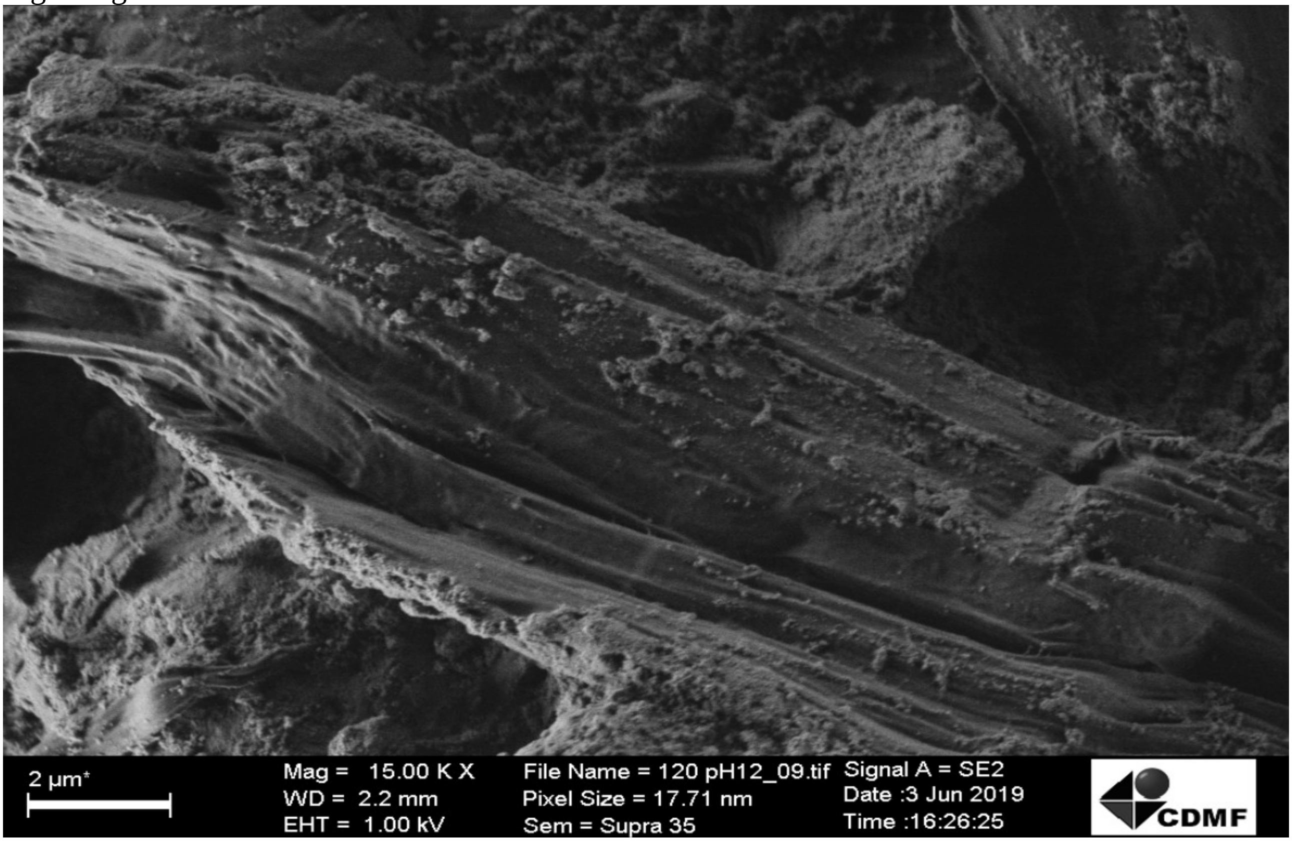

Figure 2g1

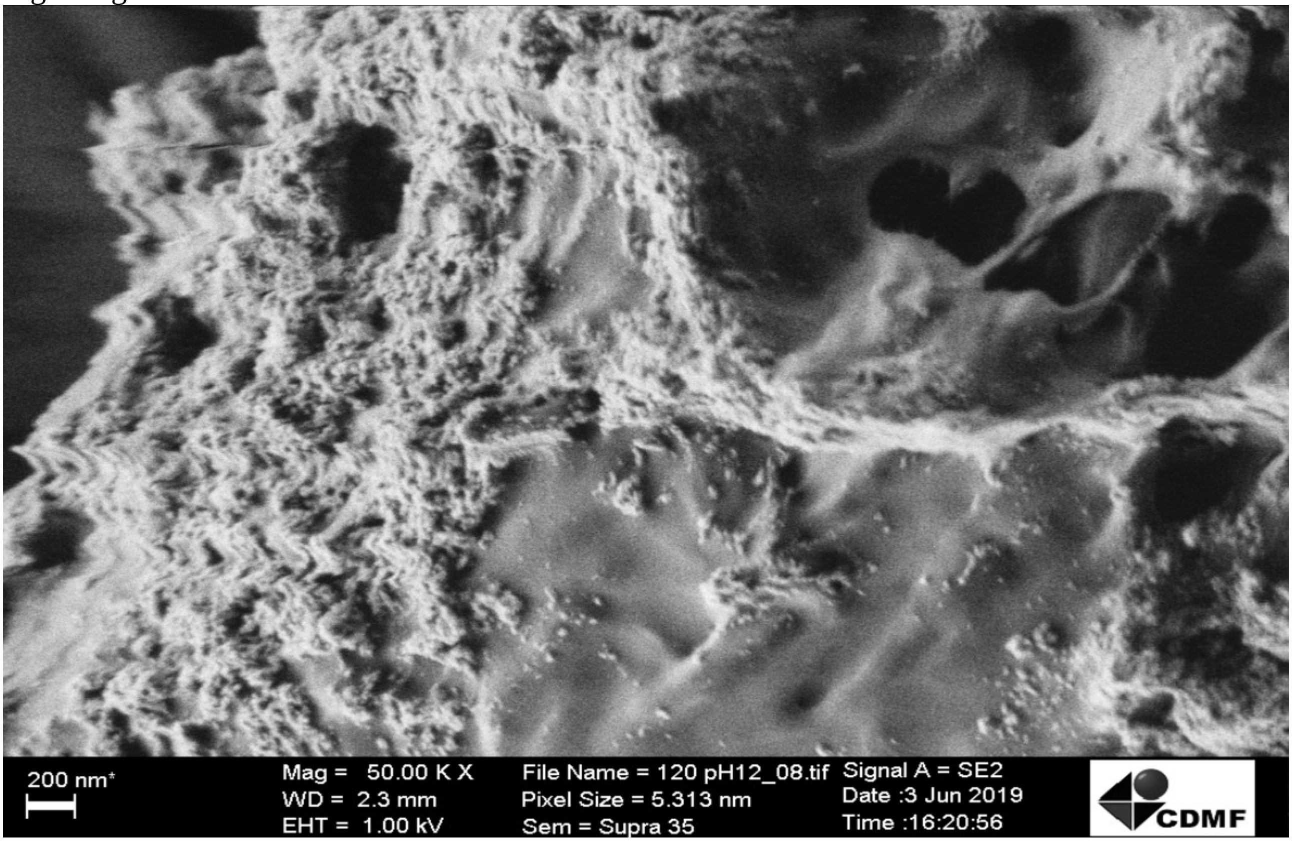

Supplement: Supplementary file 1 — Supplementary Information. [file 41598_2022_7200_MOESM1_ESM.pdf]
